# Restrictive intraoperative fluid optimisation algorithm improves outcomes in patients undergoing pancreaticoduodenectomy: A prospective multicentre randomized controlled trial

**DOI:** 10.1371/journal.pone.0183313

**Published:** 2017-09-07

**Authors:** Laurence Weinberg, Damian Ianno, Leonid Churilov, Ian Chao, Nick Scurrah, Clive Rachbuch, Jonathan Banting, Vijaragavan Muralidharan, David Story, Rinaldo Bellomo, Chris Christophi, Mehrdad Nikfarjam

**Affiliations:** 1 Department of Surgery, Austin Hospital, The University of Melbourne, Heidelberg, Victoria, Australia; 2 Anaesthesia and Perioperative and Pain Medicine Unit, The University of Melbourne, Parkville, Victoria, Australia; 3 Department of Anaesthesia, Austin Hospital, Heidelberg, Victoria, Australia; 4 Statistics and Decision Analysis Academic Platform, The Florey Institute of Neuroscience and Mental Health, Melbourne Brain Centre, Heidelberg, Victoria, Australia; 5 Department of Anaesthesia, Box Hill Hospital, Box Hill, Victoria, Australia; 6 Intensive Care Unit, Austin Hospital, Heidelberg, Victoria, Australia; Cardiff University, UNITED KINGDOM

## Abstract

We aimed to evaluate perioperative outcomes in patients undergoing pancreaticoduodenectomy with or without a cardiac output goal directed therapy (GDT) algorithm. We conducted a multicentre randomised controlled trial in four high volume hepatobiliary-pancreatic surgery centres. We evaluated whether the additional impact of a intraoperative fluid optimisation algorithm would influence the amount of fluid delivered, reduce fluid related complications, and improve length of hospital stay. Fifty-two consecutive adult patients were recruited. The median (IQR) duration of surgery was 8.6 hours (7.1:9.6) in the GDT group vs. 7.8 hours (6.8:9.0) in the usual care group (p = 0.2). Intraoperative fluid balance was 1005mL (475:1873) in the GDT group vs. 3300mL (2474:3874) in the usual care group (p<0.0001). Total volume of fluid administered intraoperatively was also lower in the GDT group: 2050mL (1313:2700) vs. 4088mL (3400:4525), p<0.0001 and vasoactive medications were used more frequently. There were no significant differences in proportions of patients experiencing overall complications (p = 0.179); however, fewer complications occurred in the GDT group: 44 vs. 92 (Incidence Rate Ratio: 0.41; 95%CI 0.24 to 0.69, p = 0.001). Median (IQR) length of hospital stay was 9.5 days (IQR: 7.0, 14.3) in the GDT vs. 12.5 days in the usual care group (IQR: 9.0, 22.3) for an Incidence Rate Ratio 0.64 (95% CI 0.48 to 0.85, p = 0.002). In conclusion, using a surgery-specific, patient-specific goal directed restrictive fluid therapy algorithm in this cohort of patients, can justify using enough fluid without causing oedema, yet as little fluid as possible without causing hypovolaemia i.e. “precision” fluid therapy. Our findings support the use of a perioperative haemodynamic optimization plan that prioritizes preservation of cardiac output and organ perfusion pressure by judicious use of fluid therapy, rational use of vasoactive drugs and timely application of inotropic drugs. They also suggest the need for further larger studies to confirm its findings.

## Introduction

Pancreaticoduodenectomy (PD) remains the primary treatment strategy for strategy peri-ampullary malignancies including pancreatic adenocarcinoma and a variety of benign conditions. It is a complex operative procedure associated with a significant physiological impact on patients requiring a long recovery period. Despite achieving relatively low peri-operative mortality in specialised centres [1,2] it continues to be associated with high peri-operative complication rates ranging between 17–50% [3,4]. With projected increases in the rates of pancreatic cancer [5], there is an increasing need to reduce complication rates associated with PD.

Inappropriate perioperative fluid intervention and likely tissue hypoperfusion and/or oedema are strongly associated with the development of postoperative complications for patients undergoing major abdominal surgery including PD [4,6–8]. Enhanced Recovery After Surgery programmes (ERAS) for patients undergoing PD have advocated a more judicious use of intravenous (IV) fluid administration [9,10]. ERAS programs for PD have been widely adopted impacting positively on length of stay, while not increasing rates of peri-operative morbidity, mortality or readmission [11]. While ERAS has been shown to be beneficial to patient outcomes when compared to usual care in patients receiving PD, no trials have examined the additional impact of an intraoperative fluid optimisation algorithm. We hypothesised that for patients undergoing PD using ERAS protocols, the additional impact of an intraoperative fluid optimisation algorithm would influence the amount of fluid delivered, reduce fluid related complications, and improve length of hospital stay. We conducted a prospective multicentre randomized controlled trial to test our hypothesis.

## Methods

The Austin Health Research and Ethics Committee approved this study (Approval number: HREC/13/Austin/30) and written informed consent was obtained from all participants. The study was retrospectively registered with the Australian New Zealand Clinical Trials Registry (ACTRN: 12616000538448). The study was conducted from September 2013 to December 2015 at four metropolitan hospitals with a dedicated hepatobiliary service.

### Key dates

Trial design and final Protocol lock: 23 Apr 2013

Submitted for Research Ethics Committee approval: 23 Apr 2013

Research Ethics Committee Approval date: 2 Sept 2013

First participant enrolled: 9 Sept 2013

Last participant enrolled: 31 Dec 2015

Project completion date: 2 Feb 2016

Participants were identified from elective surgery waiting lists and included adult patients undergoing elective PD. We excluded the following patients: age less than 18 years, pregnancy, pre-operative coagulopathy, renal impairment (creatinine >250umol/L), chronic liver disease (Child Pugh classification), American Society Anaesthesiology physical status > class IV, and patients undergoing distal, central or total pancreatectomy or pancreatic enucleation.

Prior to randomisation, in keeping with standard hospital practices at all centres, all patients regardless of their diagnosis being considered for PD underwent preoperative multidisciplinary team assessment where surgeon, anaesthetist, radiologist, oncologist, nutritionist and allied health professionals ensured patients were optimized for surgery. This included a haematology led multimodal perioperative haemoglobin optimization program based on the National Blood Authority of Australia’s patient blood management initiatives to optimize preoperative red cell mass, minimize perioperative blood loss and tolerate postoperative anaemia [12]. Patients randomized to the ERAS group received standard ERAS care (usual care group). Patients randomized to the GDT group received standard ERAS care in addition to a surgery-specific fluid optimisation algorithm.

### Standardised ERAS protocol

Participants were fasted for six hours for solids and two hours for clear fluids. Intravenous (IV) fluid loading prior to induction of anaesthesia was prohibited. Unless contraindicated, intrathecal morphine analgesia (300-400ug) was inserted at a lumbar spinal level prior to induction of anaesthesia. Patients with morphine allergy received epidural analgesia via a low thoracic needle inserted at T8/9 or T9/10 level. Anaesthesia was induced with propofol (1-3mg/kg IV), fentanyl (1-3ug/kg IV) and a non-depolarizing neuromuscular blocker. Intraoperatively, participants received dexamethasone (8mg IV), clexane (40mg subcutaneously) and paracetamol (1g IV). Antibiotic prophylaxis included ceftriaxone (1g IV), ampicillin (1g IV) and metronidazole (500mg IV). Intraoperative monitoring included continuous electrocardiography, pulse oximetry, capnography, invasive blood pressure, central venous pressure, pulse pressure variation, urine output and core body temperature. Maintenance of anaesthesia was achieved using sevoflurane or desflurane in 50% oxygen: 50% air ratio titrated to a bispectral index (BIS) of 40 to 60. Remifentanil (0.1–0.3ug/kg/min IV) was started after induction of anaesthesia and ceased during surgical closure of the wound. Thirty minutes prior to wound closure, participants receiving epidural analgesia were loaded with ropivicane 0.2% (10mL via the epidural catheter) followed by an epidural infusion at 10mL/hr. All other participants received fentanyl (20-100ug/mL) at the discretion of the attending anaesthetist.

### Intraoperative fluid management and use of vasoactive medications

All participants had blood pressure measured directly from a 20G arterial line catheter inserted prior to induction of anaesthesia. Central venous pressure was recorded directly and continuously from a central venous catheter. The FloTrac^™^ catheter (FloTrac System 4.0, Edwards Lifesciences, Irvine, CA, USA) was connected directly to the arterial line and then connected to the EV1000 haemodynamic monitor. The monitor displayed stroke volume index and cardiac index, which are derived variables calculated by the Frotrac Catheter using the arterial pressure wave form. The monitor also displayed the stroke volume variation (derived variable calculated from the maximum, minimum and mean stroke volume over a respiratory cycle), and the systemic vascular resistance that was calculated from mean arterial pressure, central venous pressure and cardiac output using standard physiologic formulae.

**Fig 1:**
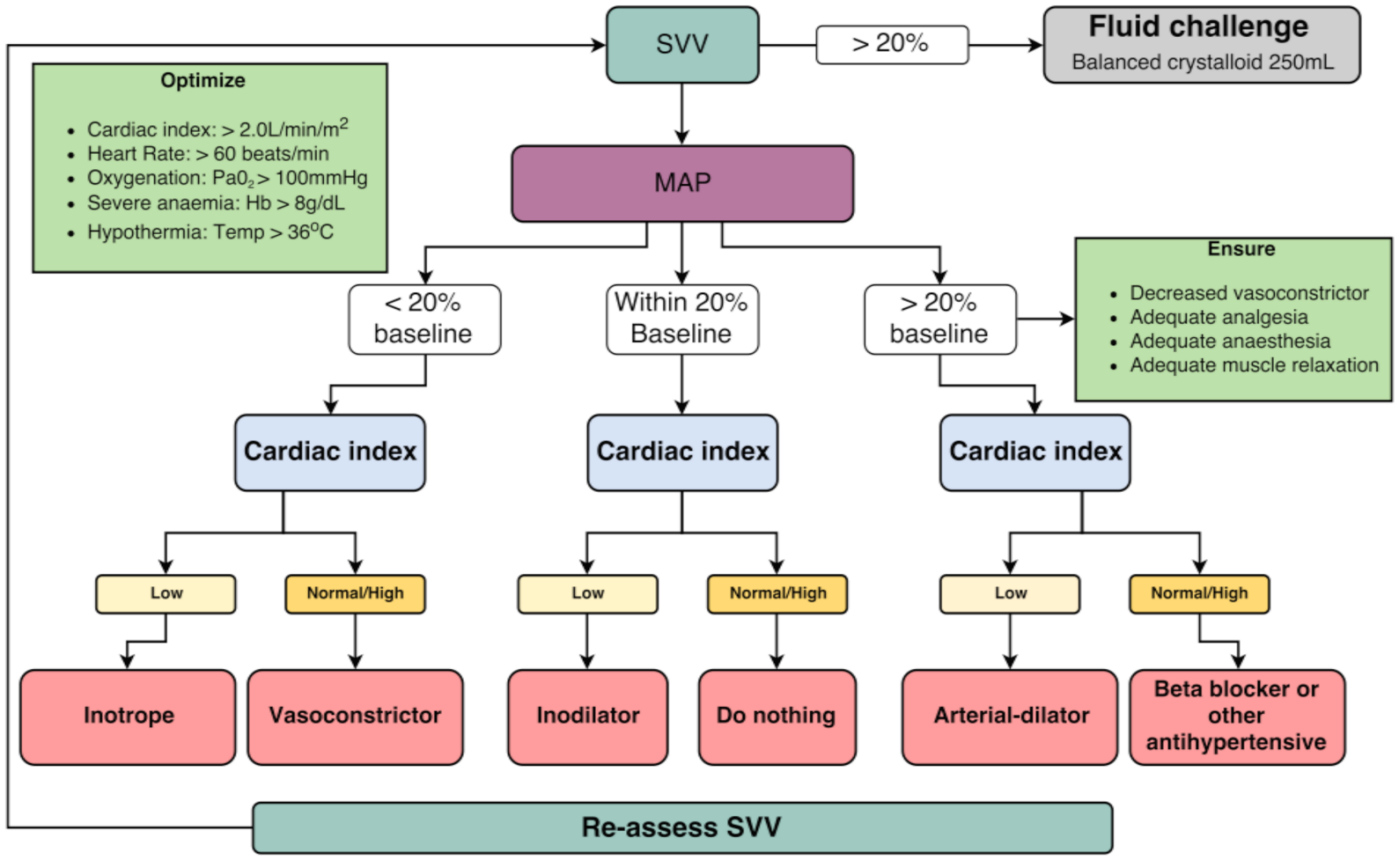
Surgery-specific cardiac output algorithm.

The arterial line pressure bag was maintained at 300 mmHg, with the sensor stopcock kept level to the phlebostatic axis, located at the fourth intercostal space at the mid-anterior-posterior diameter of the chest wall (corresponding to the right atrium). Patients in the usual care group had the EV1000 monitor concealed with an opaque screen with all alarms silenced. Fluid management and use of any vasoactive medication was guided only by routine cardiovascular monitoring at the discretion of the attending anaesthetist. For participants in the GDT group, fluid intervention and use of vasoactive medications were guided by an intraoperative fluid optimisation algorithm (Fig 1) and based on physiological parameters from the EV1000 haemodynamic monitor in addition to conventional haemodynamic monitoring. A stroke volume variation of >20% was used as a target for fluid intervention. At the end of surgery, all haemodynamic information from the EV1000 monitor was downloaded from all participants. For all participants, the only available crystalloids solutions were Hartmann’s solution or Plasma-Lyte 148 (Baxter Healthcare, Toongabie, New South Wales, Australia). The only colloid solutions were 4% or 20% Albumex (CSL Behring, Broadmeadows, Victoria, Australia).

Postoperatively, all participants were admitted to the intensive care unit for at least 24 hours, and then discharged to a dedicated hepatobiliary surgical ward under a multidisciplinary team of surgeon, perioperative physician, and pain clinician. Postoperative analgesia was optimized twice daily by a dedicated acute pain service. Fentanyl patient controlled analgesia was initiated in the ICU (20ug bolus, five-minute lock out), until oral intake resumed, after which participants receive oral oxycodone (10-20mg every 4 hours). All participants received a low dose ketamine infusion (0.05–0.1mg/kg/hr) for 24 postoperative hours. Epidural analgesia was ceased at 48–72 hours postoperatively. All participants received strict paracetamol (1g QID) for 48 hours and proton pump inhibitor continued for two weeks post discharge. Rescue analgesia consisted of non-steroidal anti-inflammatory drugs (ketorolac 30mg IV 8 hourly) or tramadol (50-100mg IV 6 hourly). Physiotherapy was delivery twice daily and antibiotic prophylaxis continued for 24 hours. Nasogastric tubes were removed the day following surgery, unless there was a greater than 300mL drainage in a 6-hour period. Clear fluids were encouraged immediately postoperatively, and liquid diet was commenced on postoperatively Day 2. The surgical drains were removed when there was no evidence of pancreatic or biliary leakage. Pancreatic enzyme supplements commenced once a soft diet was tolerated. Strict serum glucose control (target of 6 to 10 mmol/L) was maintained by use of an insulin sliding scale. The indwelling urinary catheters was removed by Day 3. Laxatives (docusate sodium 200mg) every twelve hours were commenced from Day 4 post surgery to achieve regular bowel motions.

### Outcomes and data collected

The primary outcome was length of hospital stay (defined as discharge from theatre to formal discharge from the acute hospital ward). The criteria for discharge were unassisted mobilisation, eating and drinking without nausea or vomiting, defaecation, satisfactory oral analgesia, and no evidence of medical or surgical complications, particularly infection. Other outcomes collected were the amount of fluid administered perioperatively, use of vasoactive medications, and development of complications. Complications were recorded by two independent clinicians as unexpected events occurring in the postoperative period until hospital discharge, and graded according to Clavien-Dindo Classification [13]. Pancreatic leaks and delayed gastric emptying were graded and classified according to the International Study Group of Pancreatic Surgery [14–16]. All other complications were defined and classified according to the European Perioperative Clinical Outcome (EPCO) definitions, based on a statement from the European Society of Anaesthesiology and the European Society of Intensive Care Medicine joint taskforce on peri-operative outcome measures [17].

Other data collected included preoperative patient characteristics, body mass index, American Society of Anesthesiologists (ASA) class, comorbidities and preoperative biochemical and haematological laboratory test results. Operative details collected included anaesthetic technique, fluid balances, intraoperative blood transfusion requirements, duration of surgery, and use of vasoactive medications (type and amount). Fluid balances were calculated by subtracting total output (urine output, blood loss, loss from drains and vomitus) from total input (all intravenous fluid intervention, parental medications or feeding, oral water intake). Third space losses were not included, as they were considered negligible. Postoperative details included detailed fluid intervention for postoperative Days 1 and 2 (type and amount), detailed fluid balances for postoperative Days 1 and 2, blood transfusion requirements, daily body weight, routine biochemistry and haematology, and drain output.

### Sample size estimation and statistical methodology

We powered the study to observe a large treatment effect (Cohen’s d = 0.8). In order to observe such effect 52 patients in total (26 per group) would provide a power of 0.8, assuming the type 1 error threshold of 0.05. Based on previous pilot data from our institution, the expected LOS in the control group was 16 days (SD = 4 days). Effects size d = 0.8 would correspond to the difference of 3.2 days, thus the expected LOS in intervention group would be 12.8 days. An independent statistician generated a computerised sequence of 52 allocation codes, 26 for each group. An independent research nurse sealed the allocation codes into sequentially numbered opaque envelopes. Randomisation was done by a central unit immediately prior to induction of anaesthesia. Statistics were done by a statistician blinded to allocation and the code was broken after analysis was completed.

Statistical analysis was performed using commercial statistical software STATA/IC v.13. Figures were constructed using Prism 7.0 GraphPad software (La Jolla, CA, USA). Results were expressed as either a median (range) or in the form of frequencies unless otherwise stated. Comparisons between categorical variables were determined by chi-square and Fisher’s exact test as appropriate. Non-categorical variables were assessed by the Mann-Whitney U test. Associations between GDT and individual outcomes were investigated using appropriate regression models: negative binomial regression models for LOS (treated as the count of days) and for total per patient count of postoperative complications, linear regression models with robust standard error estimation for fluid outcomes, and logistic regression models for the use of individual vasoactive drugs. Duration of surgery was used as a marker of complexity for surgery and added as a covariate in all analyses performed. Corresponding associations are summarized as appropriate effect sizes with 95% confidence intervals (CIs). The difference between groups with highest grade of complications was estimated using Wilcoxon-Mann-Whitney Generalised odds ratio and corresponding 95%CI, A p value of 0.05 was chosen as the threshold to indicate statistical significance. In order to preserve Type I error, a p value of less than 0.01 was considered statistically significant for IV fluid and vasoactive drugs where multiple outcomes were being tested. The study was reported in accordance with the CONSORT Guidelines for reporting randomised trials [18].

## Results

**Fig 2:**
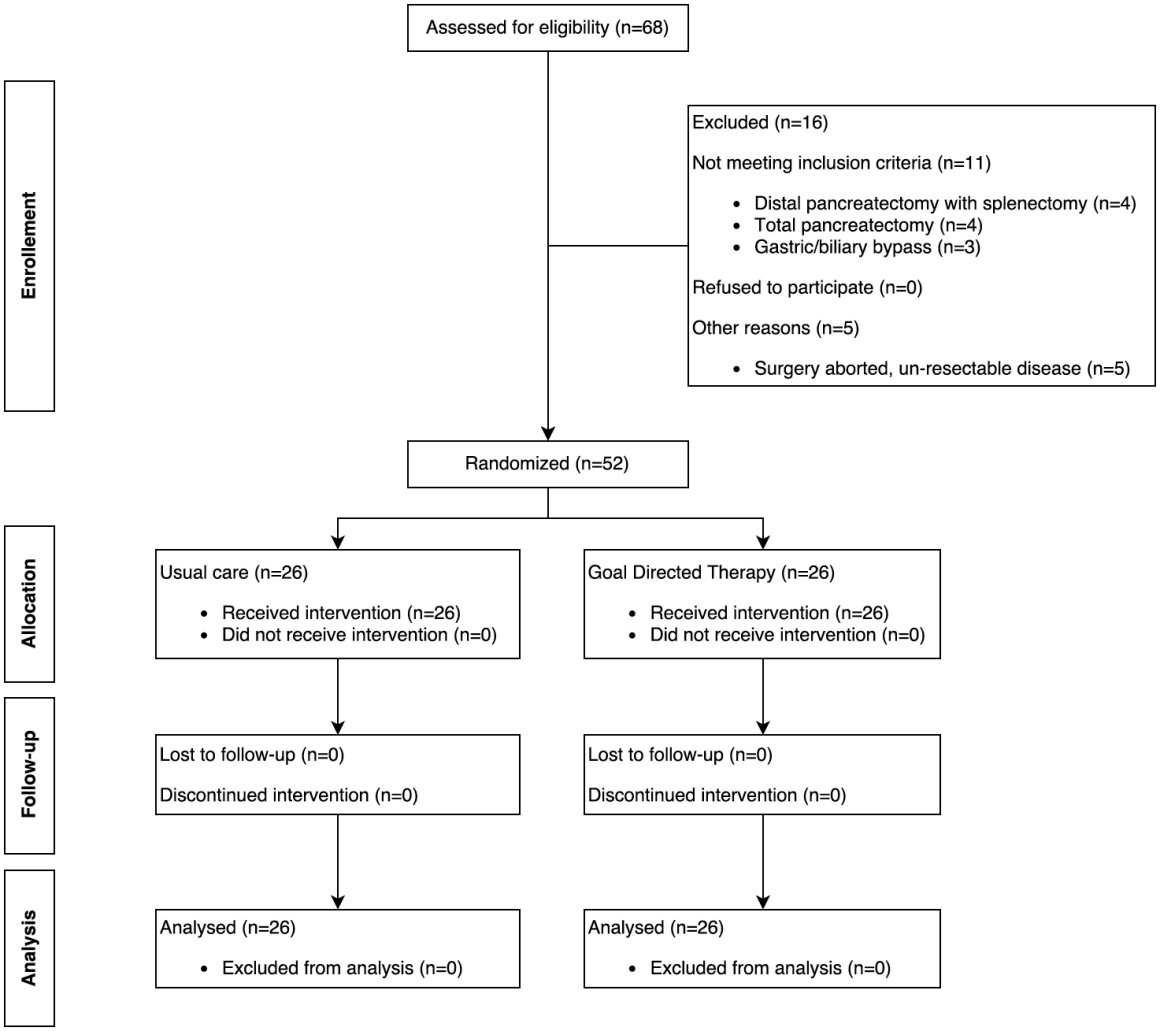
Consort diagram.

Sixty-eight participants were screened for eligibility, 16 patients had changes to planned operative interventions and were excluded (distal pancreatectomy with splenectomy: 4, total pancreatectomy: 4, surgery aborted due to unresectable disease: 5, and palliative gastric/biliary bypass: 3). Twenty-six participants were randomized to GDT and twenty-six participants to usual care (Fig 2). There were no violations or breaches of the study or ERAS protocols. Baseline characteristics, co-morbidities, and preoperative biochemical and haematological results are summarised in Table 1. The median (IQR) age was 61 years (53,72) in the GDT group and 68 years (54,75) in the usual care group respectively. Nineteen patients (73%) in the GDT group received intrathecal morphine vs. 18 patients (69%) in the usual care group, the remaining patients received epidural anaesthesia. Median (IQR) duration of surgery was 8.6 (7.1,9.6) in the GDT group vs. 7.8 hours (6.8,9.0) in the Usual care group (p = 0.2). Cancer was the most common indication for surgery in both groups.

**Table 1:** Characteristics of patients undergoing pancreaticoduodenectomy with and without goal directed therapy. Data presented as median (interquartile range) or number (proportion).

	GDT group (n = 26)	Usual care group (n = 26)
**Characteristics**		
Age (years)	61 (53:72)	68 (54:75)
Male:Female	15:11	14:12
BMI (kg/m^2^)	27 (23:31)	28 (24:31)
ASA Class I-II	7 (27%)	7 (27%)
ASA Class ≥ III	19 (73%)	19 (73%)
Diabetes	7 (27%)	11 (42%)
COPD	4 (15%)	2 (8%)
Hypertension	4 (15%)	3 (12%)
Ischemic Heart Disease	1 (4%)	2 (8%)
PVD	2 (8%)	1 (4%)
Malignancy	25 (96%)	25 (96%)
**Preoperative bloods**		
Hb (g/L)	141 (130:148)	135 (125:145)
WCC (x10^^9^/L)	7.1 (5.7:8.6)	7.2 (5.8:9.9)
Platelets (x10^^9^/L)	234 (185:296)	223 (199:303)
Albumin (g/L)	40 (37:45)	37 (31:41)
Bilirubin (μmol/L)	10 (7:15)	12 (8:42)
Urea (mmol/L)	5.9 (4.4:6.7)	6.2 (4.7:7.6)
Creatinine (μmol/L)	69 (59:86)	73 (59:98)
eGFR (mL/min/1.73m^2^)	90 (80:90)	82 (66:91)

ASA–American society of anesthesiologists; BMI–body mass index; WCC–white cell count, COPD–Chronic obstructive pulmonary disease, PVD–Peripheral vascular disease. Missing values; Hb 1, WCC 1, Platelets 1, Albumin 1, Bilirubin 3, Urea 1, eGFR 1.

### Primary endpoint: Length of stay

Median (IQR) length of hospital stay was significantly shorter in the GDT group: 9.5 days (IQR: 7.0, 14.3) compared to the Usual care group: 12.5 days (IQR: 9.0, 22.3) with an Incidence Rate Ratio (IRR) of 0.64 (95% CI 0.48, 0.85), p = 0.002. The median (IQR) for length of stay for each of the four recruiting hospitals was 12 days (9:16), 9 days (7.5:15), 11 days (6:23), and 10.5 days respectively (7.25:16.25). There were no differences between the length of stay between the four hospitals (p = 0.465: Kruskal-Wallis test ANOVA).

### Secondary endpoints

#### Intraoperative fluids and vasoactive medications

Detailed intraoperative fluid intervention, vasoactive medications and hospital length of stay are summarized in Table 2. Median (IQR) intraoperative fluid balance was lower in the GDT group compared to the usual care group (p<0.0001). Crystalloid use was significantly lower in the GDT group (p<0.0001). There were no significant differences in the use of intraoperative colloid or blood products. Three patients (12%) in the GDT group received intraoperative metaraminol vs. 23 patients (88%) in the usual care group (p<0.0001). Eleven patients (42%) received intraoperative ephedrine in the GDT group vs. 7 patients (27%) in the usual care (p = 0.125). Twenty-four patients (92%) in the GDT group received intraoperative noradrenaline vs. 8 patients (31%) in the usual care group (p<0.0001). Similarly, the use of dopamine and/or dobutamine was significantly higher in the GDT group compared to the usual care group: 12 patients (46%) vs. 1 patient (4%) (p = 0.007). Use of intraoperative beta-blockers was similar between groups.

**Table 2:** Intraoperative fluid intervention, vasoactive drug administration, operative factors, regional anaesthesia and length of stay in patients undergoing pancreaticoduodenectomy with and without goal directed therapy (GDT). Data presented as median (interquartile range) or number (proportion).

	GDT group (n = 26)	Usual care group (n = 26)	Effect size (95% CI)	p value
**Fluid intervention**				
Crystalloid (mL)	1750 (1000:2100)	4000 (2313:4206)	-1787 (-2453:-1121)^a^	<0.0001
Colloid (mL)	200 (0:500)	200 (0:500)	-94 (-369:182)^a^	0.499
Blood products	0	1 (4%)	Not estimable^b^	>0.999
Total fluid (mL)	2050 (1313:2700)	4088 (3400:4525)	-1881 (-2490:-1271)^a^	<0.0001
Total fluid (mL/kg/hr)	3.2 (2.2:3.9)	6.8 (5.4:8.6)	-3.40 (-4.37:-2.44)^a^	<0.0001
Urine output (mL)	605 (310:1128)	669 (273:948)	25 (-313:364)^a^	0.880
Blood loss (mL)	200 (138:363)	400 (200:550)	-135 (-284:15)^a^	0.076
Fluid balance (mL)	1005 (475:1873)	3300 (2474:3874)	-1808 (-2469:-1148)^a^	<0.0001
**Vasoactive drugs**				
Any vasoactive drug given	26 (100%)	25 (96%)	Not estimable^b^	>0.999
Metaraminol	3 (12%)	23 (88%)	0.02 (0.00:0.10)^b^	<0.0001
Ephedrine	11 (42%)	7 (27%)	2.69 (0.76:9.49)^b^	0.125
Phenylephrine	0	2 (8%)	0.20 (0:2.42)^b^	0.2
Noradrenaline	24 (92%)	8 (31%)	28.07 (4.90:160.71)^b^	<0.0001
Beta-blockers	5 (19%)	4 (15%)	1.37 (0.32:5.97)^b^	0.672
Dopamine/Dobutamine	12 (46%)	1 (4%)	26.17 (2.46:277.96)^b^	0.007
**Operative factors**				
Duration of surgery (hours)	8.6 (7.1:9.6)	7.8 (6.8:9.0)	0.56 (-0.31:1.43)^a^	0.2
**Length of hospital stay**				
Hospital stay (days)	9.5 (7.0:14.3)	12.5 (9.0:22.3)	0.64 (0.48:0.85)^c^	0.002

^a^Effect size reported as average difference with robust 95%CI

^b^Effect size reported as odds ratio

^c^Effect size reported as incidence rate ratio

Bonferonni corrected threshold for statistical significance: p = 0.00625 for fluids, and 0.0071 for vasoactive drugs

#### Postoperative fluids

Details of postoperative fluid interventions are summarized in Table 3. Median (IQR) Postoperative Day 1 fluid balance was 1661mL (1253, 2041) in the GDT group vs. 1177mL (704, 1725) in the usual care group (p = 0.178). Postoperative Day 2 fluid balances were also similar between groups: 334mL (-426, 884) in the GDT group vs. 212mL (-767, 636) in the usual care group (p = 0.239). No statistically significant differences in the volumes of crystalloid or colloid fluids administered between the groups on both postoperative Day 1 and Day 2 were observed.

**Table 3:** Postoperative fluid intervention in patients undergoing pancreaticoduodenectomy with and without goal directed therapy (GDT). Data presented as median (interquartile range) or number (proportion).

	GDT group (n = 26)	Usual care group (n = 26)	Effect size (CI)	p value
**Day 1**				
Crystalloids (mL)	2330 (2030:3119)	2627 (1908:3072)	-59 (-552:435)^a^	0.813
Colloids (mL)	0 (0:350)	0 (0:750)	-203 (-430:24)^a^	0.078
Blood products	0	0		
Total IV fluid (mL)	2466 (2045:3323)	2946 (2199:3481)	-262 (-819:296)^a^	0.35
Fluid balance (mL)	1661 (1253:2041)	1177 (704:1725)	331 (-156:818)^a^	0.178
**Day 2**				
Crystalloids (mL)	1544 (1376:2151)	1900 (1544:2259)	-226 (-511:59)^a^	0.118
Colloids (mL)	0 (0:0)	0 (0:0)	22 (-23:67)^a^	0.326
Blood products	0	2 (8%)	0.32 (0:4.50)^b^	0.4
Total IV fluid (mL)	1570 (1376:2151)	1900 (1594:2259)	-247 (-544:51)^a^	0.102
Fluid balance (mL)	334 (-426:884)	212 (-767:636)	351 (-241:944)^a^	0.239

^a^Effect size reported as average difference with robust 95%CI

^b^Effect size reported as odds ratio

#### Postoperative complications

**Fig 3:**
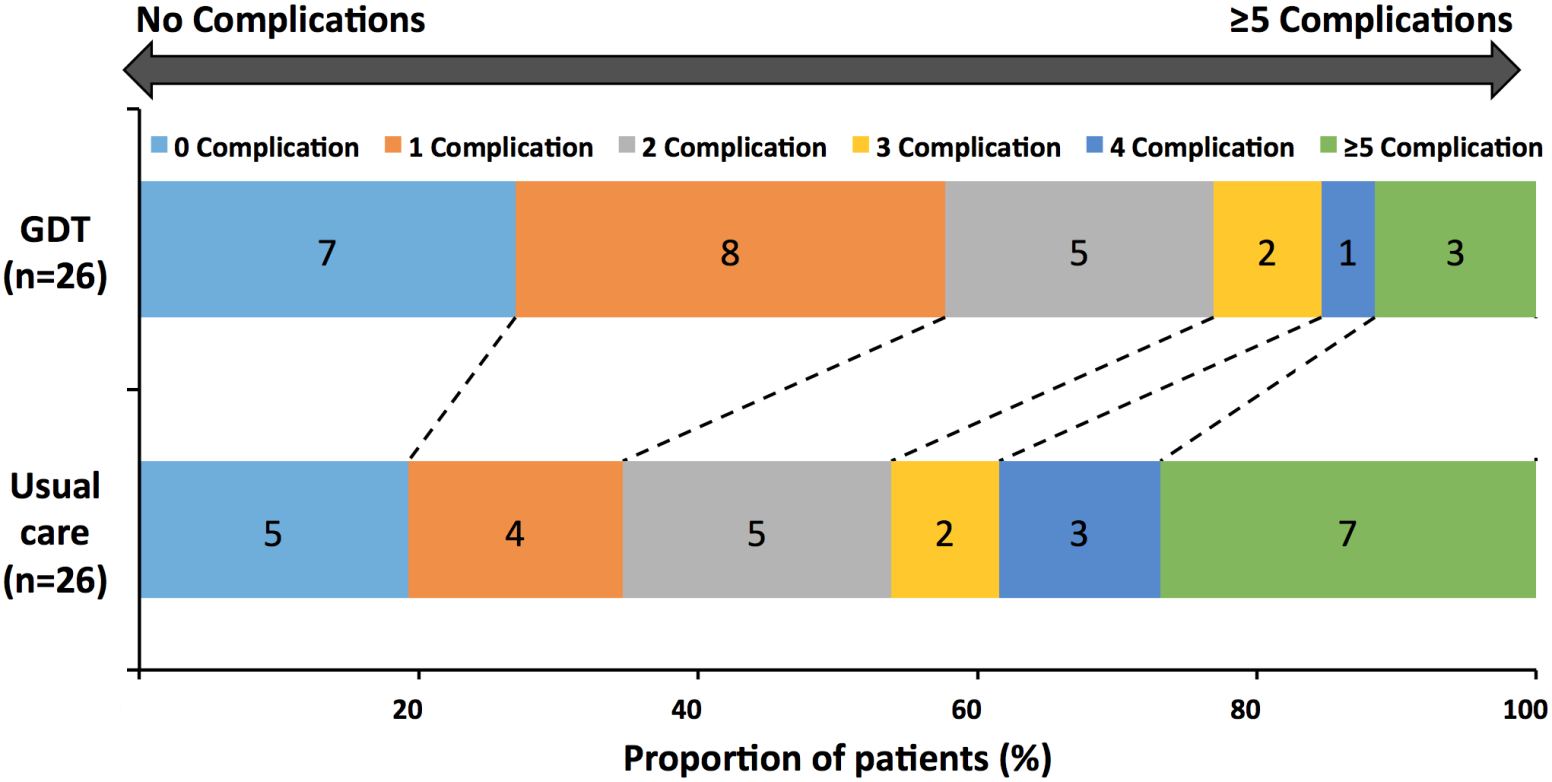
Modified Rankin scale showing the proportion of participants in the usual care and goal directed therapy (GDT) groups with complications.

Details of postoperative complications are summarized in Table 4. Postoperative complications were common and occurred at similar frequencies amongst the GDT (73%) and usual care (81%) groups (p = 0.179). Total number of complications per patient were significantly lower in GDT group (44) than with usual care (92): IRR: 0.41 (95%CI 0.24, 0.69) p = 0.001 (Fig 3). The majority of complications were graded as Clavien-Dindo Class 1 and 2. Assessment of most severe complications demonstrated no significant differences between the GDT group and usual care group (p = 0.414). Postoperative pancreatic fistula occurred in 2 patients (8%) in the GDT group vs. 5 patients (19%) in the usual care group (p = 0.191). Three patients (12%) in the GDT group developed delayed gastric emptying vs. 6 patients (23%) in the usual care group (p = 0.213). Patients in the usual care group were significantly more likely to receive blood transfusion: GDT (nil) vs. usual care (35%) (p = 0.0005). Patients in the usual care group were significantly more likely to develop electrolyte derangements: GDT (27%) vs. usual care (62%) (p = 0.012). A difference between the proportions of cardiorespiratory complications in the usual care group (54%) compared to GDT (27%) was noticed, which did not achieve statistical significance (p = 0.066). The rest of the complications were similar between the groups. No significant differences between the groups in return to theatre (p = 0.521), or return to ICU (p>0.999) were observed.

**Table 4:** Summary of complications in patients undergoing pancreaticoduodenectomy with and without goal directed therapy. Data presented as number (proportion).

	GDT group (n = 26)	Usual care group (n = 26)	Effect size (CI)	p value
**Patients with complications**	19 (73%)	21 (81%)	0.34 (0.07–1.64)^e^	0.179
**Number of complications (total)**	44 (1.69)	92 (3.54)	0.41 (0.24:0.69)^f^	0.001
**Clavien-Dindo Classification (most severe)**			1.28 (0.70:2.33)^g^	0.414
I	6 (23%)	5 (19%)		
II	10 (38%)	13 (50%)		
III	3 (12%)	0		
IV	0	3 (12%)		
V	0	0		
**Complication types**				
Wound infection	5 (19%)	6 (23%)	0.76 (0.19:2.95)^e^	0.687
Superficial surgical site infection	4 (15%)	4 (15%)	0.83 (0.17:3.95)^e^	0.812
Deep surgical site infection	1 (4%)	2 (8%)	0.31 (0.02:4.33)^e^	0.387
Sepsis	2 (8%)	3 (12%)	0.61 (0.09:4.14)^e^	0.613
Postoperative pancreatic fistula^a^	2 (8%)	5 (19%)	0.30 (0.05:1.82)^e^	0.191
Grade A	1 (4%)	2 (8%)	0.39 (0.03:4.92)^e^	0.463
Grade B	1 (4%)	2 (8%)	0.51 (0.04:6.24)^e^	0.601
Grade C	0	1 (4%)	Not estimable ^e^	-
Delayed gastric emptying	3 (12%)	6 (23%)	0.37 (0.08:1.77)^e^	0.213
Bile leak^b^	1 (4%)	0	Not estimable	-
Cardiorespiratory complications	7 (27%)	14 (54%)	0.33 (0.10:1.07)^e^	0.066
Acute respiratory distress syndrome	0	1 (4%)	1 (0:39.00)^e^	>0.999
Pneumonia	1 (4%)	1 (4%)	0.62 (0.03:12.56)^e^	0.758
Pulmonary atelectasis	1 (4%)	3 (12%)	0.20 (0.02:2.45)^e^	0.210
Pulmonary congestion	3 (12%)	3 (12%)	0.80 (0.14:4.70)^e^	0.805
Cardiogenic pulmonary oedema	0	3 (12%)	0.07 (0:1.08)^e^	0.057
Arrhythmia	2 (8%)	3 (12%)	0.56 (0.08:3.87)^e^	0.558
Acute pancreatitis^c^	1 (4%)	0	Not estimable^e^	>0.999
Gastrointestinal bleed	0	2 (8%)	1 (0:39.00)^e^	>0.999
Acute kidney injury	4 (15%)	4 (15%)	0.83 (0.17:3.96)^e^	0.813
Delirium	2 (8%)	7 (27%)	0.17 (0.03:1.03)^e^	0.054
Ischaemic hepatitis	0	1 (4%)	1 (0:39.00)^e^	>0.99
Nausea and vomiting	2 (8%)	1 (4%)	1.23 (0.08:18.02)^e^	0.878
Electrolyte disturbances	7 (27%)	16 (62%)	0.21 (0.06:0.71)^e^	0.012
Hypokalaemia	4 (15%)	7 (27%)	0.50 (0.12:2.02)^e^	0.330
Hyponatremia	0	3 (12%)	0.29 (0:2.73)^e^	0.289
Hypomagnesemia	1 (4%)	1 (4%)	1.04 (0.06:18.37)^e^	0.979
Hypophosphatemia	0	2 (8%)	0.66 (0:6.39)^e^	0.714
Hyperkalaemia	1 (4%)	2 (8%)	0.45 (0.04:5.58)^e^	0.537
Hypernatremia	1 (4%)	1 (4%)	1.04 (0.06:18.37)^e^	0.979
Endocrine abnormalities	3 (12%)	4 (15%)	0.55 (0.10:2.99)^e^	0.486
Drug reaction	2 (8%)	0	0.71 (0.11:N/A)^e^	>0.999
Refractory analgesia	1 (4%)	4 (15%)	0.21 (0.02:2.09)^e^	0.182
Other^d^	1 (4%)	4 (15%)	0.15 (0.01:1.69)^e^	0.126
Required blood transfusion	0	9 (35%)	0.04 (0:0.29)^e^	0.0005
Return to theatre	1 (4%)	4 (15%)	0.44 (0.04:5.44)^e^	0.521
Return to ICU	0	2 (8%)	1 (-0:39.00)^e^	>0.999

All complications defined by European Perioperative Clinical Outcome definitions^17^ except:

^a^International Study Group of Pancreatic Fistula (ISGPF)

^b^Presence of bile in the drainage fluid that persisted on postoperative day 4

^c^Elevations in serum lipase > 3× normal laboratory reference range

^d^Urinary tract infection 2, Foot drop 1, Fluid overload 1, Fall 1

^e^Effect size reported as odds ratio

^f^Effect size reported as incidence rate ratio

^g^Effect size reported as generalised odds ratio

## Discussion

### Key findings

This is a multicentre randomised controlled trial in patients undergoing PD to compare usual care based on ERAS principles or GDT using a cardiac output guided haemodynamic algorithm in addition to ERAS principles. We found that GDT-guided intra-operative treatment was associated with a less positive fluid balance, decreased administration of intraoperative fluids, greater use of intraoperative vasoactive drug infusions, a decreased number of complications, decreased administration of red cells, and shorter length of hospital stay.

### Relationship with previous studies

The demographic and clinical features of patients in this study are consistent with other studies of this operation [19,20]. Moreover, complications in the usual care arm are similar to those reported in other high volume tertiary centres [21,22]. The beneficial role of ERAS after pancreatic surgery has been established in a recent systematic review [10], and has led to the implementation of specific ERAS guidelines [23]; however, there have been inconsistent findings when assessing the optimal intraoperative fluid regime for PD [19,24,25]. In this regard, despite ERAS protocols [9,10], we observed that usual care patients received almost twice the volume of fluid intraoperatively, when compared to their GDT group counterparts. Moreover, while it has been established that ERAS protocols are able to reduce length of stay in patients undergoing uncomplicated PD [26], our findings highlight the additional benefit of GDT in improving patient outcomes when combined with a standardized ERAS programme.

Our findings also concur with other studies demonstrating an association between higher postoperative fluid balances in high-risk surgery and increased requirements for blood transfusion [27]. In this regard, patients in our study did not differ at baseline in terms of age, gender or preoperative haemoglobin, all of which are considered independent predictors of blood transfusion [28,29]. Thus, our observations further emphasise the additional impact a more liberal fluid therapy can have on haemodilution and blood transfusion requirements [30]. An increased prevalence of electrolyte disturbances, notably hypokalaemia and hyponatremia was also observed in the usual care group where fluid administration was liberal [31], a finding supported by other studies comparing fluid liberal to fluid restrictive regimens [32–34].

In patients receiving PD, we recently reported that restrictive perioperative fluid intervention and negative cumulative fluid balance were associated with fewer complications and shorter length of hospital stay [8]. Similar to these findings, fluid practices in the usual care group in the present study contradict several clinical guidelines, ERAS recommendations and reviews, which reinforce and endorse the benefits associated with a restrictive or “net-even” approach to fluid therapy [35–39]. Finally, our findings support those reported in the “OPTIMISE” Trial, where GDT was associated with a clinical benefit for patients undergoing small bowel surgery with or without pancreas surgery [40]. They are also consistent with a recent meta-analysis that concluded that goal-directed therapy reduces length of hospital stay and complications [40].

### Study implications

Our findings imply that, in patients undergoing PD, the benefits of GDT may be not only related to the volume of fluid infused intraoperatively, but to how and when fluid therapy is administered, and how and when vasoactive medications are introduced. The effects of fluid therapy combined with adrenergic and vasoactive therapy has not been formally evaluated in human clinical trials. In an animal model of fluid kinetics it was reported that adrenergic alpha_1_-receptors with vasoactive drugs accelerated, while beta_1_-receptors retarded the distribution and elimination of fluid [41]. Other kinetic models in animals have shown than low dose phenylephrine therapy may slow down the distribution of fluid from the plasma to the interstitial fluid space, thereby preventing hypovolemia [42,43]. It’s plausible that both the higher use of noradrenaline and dopamine used in the GDT group in our study preserved plasma volume, offsetting the requirements for fluid intervention. Volume expansion with fluids in combination with vasoactive therapy is poorly understood due to the confounding effects of anaesthesia, surgery and patient positioning on vaso- and venodilatation, arterial pressure, cardiac contractility, and activation of the renin-aldosterone hormonal axis. Finally, the decreased administration of red cells seen with GDT may have long-term benefits as perioperative blood transfusion has been associated with reduced survival in patients with pancreatic cancer undergoing surgical resection [44].

### Strengths and limitations

There are several strengths to this study. First, it is the largest multicentre randomised trial of a haemodynamic management in PD patients receiving an ERAS protocol. All haemodynamic variables measured from the Flotrac device were assessed invasively and were not amenable to ascertainment bias or derivation. Moreover, the cardiac output haemodynamic algorithm used was pragmatic, and non-prescriptive with regards to type of fluid or specific class of vasoactive drug. This flexibility allowed anaesthetists to prescribe therapies they were most familiar with, whilst taking into consideration the patient’s baseline physiological state, and targeting appropriate haemodynamic goals according to age and co-morbidity. In addition, and in contrast to conventional recommendations, we considered a SVV of 20% as a clear cut off value for fluid intervention. We chose such value because SVV may be inconclusive between 9% and 13% in approximately 25% of patients during general anaesthesia [45,46]. To our knowledge, ours is the highest SVV target that used for a major surgery related GDT protocol, and is more fluid restrictive than most previous conventional GDT protocols [47–53]. Finally, the outcomes used to assess the intervention were robust, quantitative, clearly different and not amenable to interpretation or ascertainment bias.

Our study also has some limitations. We did not collect information on pancreatic duct size, texture of the pancreas, number of lymph nodes retrieved or surgical complexity. However, the focus of this study was on the association of non-surgical factors with patient outcomes. Moreover, we used multivariable statistical analysis to adjust for duration of surgery as a marker of complexity, a confounder that could impact on fluid intervention and postoperative outcomes. As all hepatobiliary surgeons and anaesthetists across all centres were part of a dedicated hepatobiliary-anaesthesia service, we did not collect outcomes of individual clinicians. Finally, this study was powered to measure differences in hospital length of stay, not postoperative complications. Whilst we showed that there were fewer total complications in the GDT group, there were no significant differences in proportion of patients with complications between groups. Clearly a much larger study is required to comprehensively answer this question. As this study was performed across four hospitals, the external validity of the study appears reasonably robust. However, larger confirmatory studies are desirable. Finally, as this study focused on patients undergoing PD, we cannot extrapolate our surgery-specific cardiac output guided algorithm to other types of complex surgeries, other scheduled (or emergency) types of operations, or to older, sicker or morbidly obese patients.

### Conclusions

The findings of this study indicate that GDT using a cardiac output guided algorithm can reduce positive fluid balance, the rate of complications, the requirement for blood transfusions and length of hospital stay after PD. Using a surgery-specific, patient-specific goal directed fluid therapy algorithm in this cohort of patients, can justify using enough fluid without causing oedema, yet as little fluid as possible without causing hypovolaemia i.e. “precision” fluid therapy. These findings support the use of a perioperative haemodynamic optimization plan that prioritizes preservation of cardiac output and organ perfusion pressure by judicious use of fluid therapy, rational use of vasoactive drugs and timely application of inotropic drugs. They also suggest the need for further larger studies to confirm its findings.

## Supporting information

S1 File Original dataset. (XLSX)

S2 File Consort checklist. (PDF)

S3 File Trial protocol. (PDF)
