# Supplementary material for: Restrictive intraoperative fluid optimisation algorithm improves outcomes in patients undergoing pancreaticoduodenectomy: A prospective multicentre randomized controlled trial
Source: PLoS One. 2017 Sep 7;12(9):e0183313. doi: 10.1371/journal.pone.0183313 (PMC5589093; doi:10.1371/journal.pone.0183313)
Supplement: S3 File — (PDF) [file pone.0183313.s003.pdf]

PROTOCOL

A Prospective Multicentre Randomized Controlled Trial of Standard Compared to Surgery Specific Goal-directed Therapy (GDT) for Patients Undergoing Pancreaticoduodenectomy

Protocol Number: 1
Version: 1
Date: 23/04/2013

Authors:
A/Prof Laurence Weinberg
Dr Mehrdad Nikfarjam

Sponsor/s: Nil

CONFIDENTIAL

This document is confidential and the property of **Austin Hospital**. No part of it may be transmitted, reproduced, published, or used without prior written authorization from the institution.

Statement of Compliance

This document is a protocol for a research project. This study will be conducted in compliance with all stipulation of this protocol, the conditions of the ethics committee approval, the NHMRC National Statement on ethical Conduct in Human Research (2007) and the Note for Guidance on Good Clinical Practice (CPMP/ICH-135/95).

TABLE OF CONTENTS

CONTENTS

Table of Contents 2

1. **Glossary of Abbreviations & Terms** 4
2. **Study Sites** 5
   - 2.1 Study Location/s 5
3. **Introduction/Background Information** 6
   - 3.1 Lay Summary 6
   - 3.2 Introduction 7
   - 3.3 Background information 7
4. **Study Objectives** 12
   - 4.1 Hypothesis 12
   - 4.2 Study Aims 12
   - 4.3 Outcome Measures 12

5. **Study Design** 12
   - 5.1 Study Type & Design & Schedule 12
   - 5.2 Standard Care and Additional to Standard Care Procedures 18
   - 5.3 Randomisation 18
   - 5.4 Study methodology 19

6. **Study Population** 20
   - 6.1 Recruitment Procedure 20
   - 6.2 Inclusion Criteria 20
   - 6.3 Exclusion Criteria 20
   - 6.4 Consent 20

7. **Participant Safety and Withdrawal** 21
   - 7.1 Risk Management and Safety 21
   - 7.2 Handling of Withdrawals 21
   - 7.3 Replacements 21

8. **Statistical Methods** 21

8.1 Sample Size Estimation & Justification 21

8.2 Power Calculations 21

Results from OpenEpi, Version 2, open source calculator--SSMean 22

8.3 Statistical Methods To Be Undertaken 22

9. Data Security & Handling 22

9.1 Details of where records will be kept & How long will they be stored 22

9.2 Confidentiality and Security 23

10. Appendix 24

11. References 24

GLOSSARY OF ABBREVIATIONS & TERMS

Abbreviation	Description (using lay language)
**Pancreaticoduodenectomy Resection**	Surgical removal of the distal half of the stomach (antrectomy), the gall bladder and its cystic duct (cholecystectomy), the common bile duct (choledochectomy), the head of the pancreas, duodenum, proximal jejunum, and regional lymph nodes. Reconstruction consists of attaching the pancreas to the jejunum (pancreaticojejunostomy) and attaching the hepatic duct to the jejunum (hepaticojejunostomy) to allow digestive juices and bile respectively to flow into the gastrointestinal tract and attaching the stomach to the jejunum (gastrojejunostomy) to allow food to pass through.<br>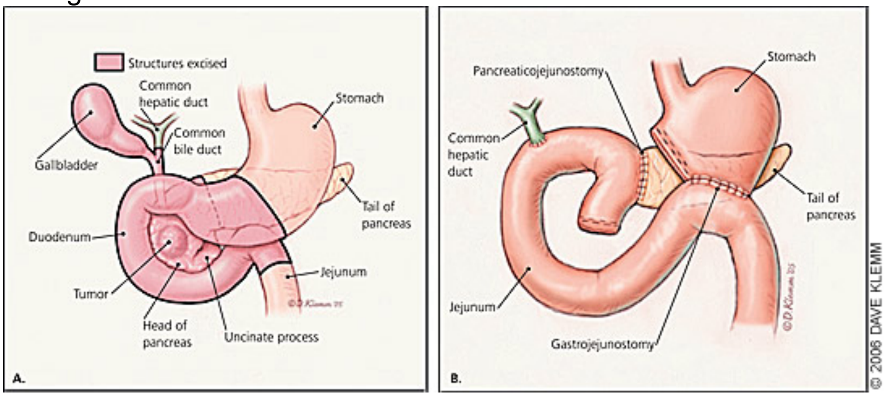
**Pylorus-sparing pancreaticoduodenectomy**	Preservation of the pylorus (preserving normal gastric emptying). Performed when the tumour does not involve the stomach and the lymph nodes along the gastric curvatures are not enlarged.
**GDT**	Goal-directed therapy
**CO**	Cardiac Output
**SVV**	Stroke Volume Variation
**SV**	Stroke Volume
**SVR**	Systemic Vascular Resistance
**GDT**	Goal-directed therapy
**HR**	Heart rate
**MAP**	Mean arterial pressure

1. Study Sites

1.1 Study Location/s

Site	Address	Contact Person	Phone	Email
Austin Hospital	Studley Road, Heidelberg, Victioria, 3084	A/Prof Laurence Weinberg	03 94965000	Laurence.Weinberg@austin.org.au
Warringal Private Hospital	216 Burgundy Street, Heidelberg, Victoria, 3084	A/Prof Laurence Weinberg	03 94965000	Laurence.Weinberg@austin.org.au
Knox Private Hospital	262 Mountain Highway Wantirna, VIC 3152	A/Prof Laurence Weinberg	03 94965000	Laurence.Weinberg@austin.org.au
Box Hill Hospital	8 Arnold Street, Box Hill, Victoria, 3128	Dr Ian Chao & Dr Clive Rachbuch	03 98953164	Ian.Chao@austin.org.au

2. Introduction/Background Information

2.1 Lay Summary

Introduction: Goal-directed therapy (GDT) is a term used to describe the use of cardiac output or similar parameters to guide intravenous fluid and inotropic therapy to ensure adequate tissue perfusion and cellular oxygenation. There is compelling evidence that fluid optimization and GDT in patients undergoing colorectal surgery leads to better outcomes, particularly in high-risk patients. However there are no current multicentre randomised controlled trials that compare standard therapy to surgery specific GDT in patients undergoing major pancreatic surgery - pancreaticoduodenectomy (Whipple's Procedure).

The aims of this study are to evaluate if the use intraoperative use of the FloTrac/Vigileo device^{TM} (FloTrac/Vigileo Version 3.02, Edwards Lifesciences, Irvine, CA, USA) improves outcomes in patients undergoing pancreaticoduodenectomy.

Hypothesis: Patients undergoing pancreaticoduodenectomy (Whipple's procedure) managed by intraoperative goal directed therapy with the Flotrac/Vigileo device^{TM} will have a shorter length of hospital stay with fewer post operative complications compared to patients managed by standard care alone.

Design: Randomised multicenter controlled clinical trial. Patients will be randomized to standard current treatment of care, or standard current treatment of care with the addition of the Flotrac/Vigileo device^{TM}. The Flotrac system will be connected to the patient prior to surgical incision and discontinued as soon as the surgery is complete.

Inclusion criteria: All patients > 18 years undergoing pancreaticoduodenectomy

Exclusion criteria

- Age less than 18 years
- Abnormal pre-operative coagulopathy: INR > 1.5, platelet count < 75 x 10^9/l
- Severe hepatic insufficiency (bilirubin > 30umol/L, ALP > 300iu/L, ALT > 50iu/L, albumin < 25g/dL, INR > 1.5)
- Severe renal impairment: serum creatinine > 250*u*mmol/l
- American Society of Anesthesiologists (ASA) physical status IV or V

Primary outcome: Length of hospital stay

Secondary outcomes

- Intraoperative haemodynamic e.g. cardiac output, blood pressure, heart rate
- Major morbidity i.e. post operative complications
- Blood transfusion requirements
- Intraoperative fluid balances (type, amount)
- Postoperative fluid balances (type, amount)
- Use of inotropes (type, amount, duration)
- Duration of ICU/HDU stay

No of recruiting sites: 4

No of patients: 52

2.2 INTRODUCTION

Goal-directed therapy (GDT) is a term used to describe the use of cardiac output or similar parameters to guide intravenous fluid and inotropic therapy to ensure adequate tissue perfusion and cellular oxygenation. There is now compelling evidence that fluid optimization and GDT in patients undergoing colorectal surgery leads to better outcomes, particularly in high risk patients. However there are no current multicentre randomised controlled trials that compare standard therapy to surgery specific GDT in patients undergoing major pancreatic surgery - pancreaticoduodenectomy (Whipples Procedure). The aims of this study are to evaluate if the use of the FloTrac/Vigileo device^{TM} (FloTrac/Vigileo Version 3.02, Edwards Lifesciences, Irvine, CA, USA) improves outcomes in patients undergoing pancreaticoduodenectomy.

The FloTrac/Vigileo system is an auto-calibrated device that analyses the arterial pressure waveform to calculate cardiac output, stroke volume, stroke volume variation and systemic vascular resistance. The Flotrac Sensor has been used in over 1 million procedures worldwide. At Austin hospital, device has been used for the last 7 years and is frequently used in high-risk patients undergoing all types of major surgery including cardiac and liver transplantation surgery. The device is a minimally invasive haemodynamic management tool that provides continuous haemodynamic information that enables the anaesthetist to make rational decisions about intraoperative and postoperative fluid and inotropic optimization. At present its use in patients undergoing pancreaticoduodenectomy is not routine as there are no studies to date that have evaluated in use in the high-risk surgical procedure.

The device automatically calculates key flow parameters every 20 seconds and recognizes and allows for adjustments in haemodynamic variables in patients undergoing major surgery. Importantly, it enables the anaesthetist to make a differential diagnosis leading to either a volume or cardiovascular intervention (preload, afterload and contractility), by providing continuous information on the patients cardiac output, stroke volume, and systemic vascular resistance.

2.3 BACKGROUND INFORMATION

Traditionally, advanced haemodynamic monitoring parameters such as stroke volume, stroke volume variation and cardiac output/cardiac index have only been available through the use of a device such as the pulmonary artery catheter. In recent years there have been a large number of new technologies available to the anaesthetist that has enabled the measurement of these parameters without the use of the pulmonary artery catheter.

There are a number of economic benefits associated with the use of advanced haemodynamic monitoring, particularly in relation to the closer monitoring and management of the patients' hydration status during major and high-risk surgery. Evidence shows that the use of this technology can facilitate improved outcomes for patients due to reduction in post-operative complications and length of stay with ultimate benefits for the health economy. This technology has been endorsed by the National Health Service (NHS) and the French Society of Anaesthesiologists (SFAR)[1,2].

The optimization of a patient's haemodynamics is one of the key factors to improving morbidity and mortality. Evidence suggests that either too little or too much fluid administration during the perioperative period can worsen tissue perfusion and oxygenation leading to organ dysfunction. Further, this impairment may not be reliably revealed by alterations in conventional haemodynamic indices such as heart rate, urine output, central venous pressure or blood pressure. Numerous investigative studies in a spectrum of patient populations (cardiovascular surgery, trauma, sepsis and other critical illnesses) have challenged the notion that these indicators accurately predict volume status[3-5]. By using the flow related parameters such as stroke volume (SV), cardiac output (CO), and markers of fluid responsiveness such as stroke volume variation (SVV) one is able more accurately infer where the patient is on their Frank Starling relationship, and thus, optimise oxygen delivery. Surgical procedures can sometimes be associated with marked, unexpected hemodynamic changes. Flow based parameters can be particularly valuable when surgical complications occur.

There is a growing body of evidence that perioperative goal-directed therapy based on flow-related haemodynamic parameters leads to reduction in complications and length of stay[6-16].

These are briefly summarised below:

In total, 40 randomized controlled trials have assessed the benefit of goal-directed therapy, spanning a wide range of high-risk surgeries:

- Of these, 37 have been included in one or more of 5 major meta-analyses[6-12]
- The total population included in these meta-analyses is 5,538 patients, of which 2,801 were randomized to GDT, and reaches back to 1985
- Trials have reported different endpoints, and as such meta-analyses of specific endpoints are based on smaller sub-populations of studies

Evidence Base

- Formal meta-analysis of effect size has shown significant benefits for GDT in:
    - Mortality (Hamilton 2010)[7]
    - Total complications (Hamilton 2010)[7]
    - Acute kidney injury (Brienza 2009)[11]
    - Pneumonia (Dalfino 2011)[10]
    - Surgical site infection (Dalfino 2011)[10]
    - Urinary tract infection (Dalfino 2011)[10]
    - Major GI complications (Giglio 2009)[8]
    - Minor GI complications (Giglio 2009)[8]
    - Length of hospital stay (Phan 2008)[12]

Why is this RCT important at Austin Hospital?

Both study investigators have completed 3 retrospective observational studies at Austin Hospital specifically evaluating postoperative complications after major hepatobiliary and pancreatic surgery.

First we measured perioperative fluid therapy, and complication rates and outcomes for patient's undergoing major elective open gastrointestinal surgery[17]. We obtained

demographic data, operative details, fluid prescription, complications, and outcomes in 100 patients. We found that most patients were male, elderly and overweight with significant pre-existing co-morbidities. Mean volume of intraoperative fluids administered was 4.2 litres. On the first and second postoperative days, the mean volume of fluid administered was 6.3 litres and 3.7 litres respectively (Table 1). Patients undergoing noncolorectal surgery (hepatobiliary/ pancreatic/esophageal) received larger fluid volumes than those undergoing colorectal procedures. Thirty-two percent of all patients had at least one major complication and 52% at least one minor complication. Major complications occurred almost 3 times more frequently in patients undergoing noncolorectal surgery compared to colorectal surgery (51% versus 22%; p = 0.004). Patients undergoing noncolorectal operations were also twice as likely to have minor complications (71.4% vs. 41.5%, p = 0.009). The most common adverse events were pulmonary oedema (21%) ileus (18%) serious sepsis (17%), pneumonia (17%), arrhythmias (14%), delirium (14%) and wound healing problems (infections 13%, anastomotic leaks 12%). In each category, the incidence was higher in the noncolorectal group. Mortality at 30 days was 2%. Age greater than 70 years, non-colorectal operations, surgery greater than 3 hours duration, and perioperative blood transfusion were all independent risk factors that predicted hospital morbidity. Hospital length of stay was more than twice as long in the non-colorectal group compared to colorectal surgery patients (30 vs. 12 days; p = 0.004). We concluded that peri-operative fluid therapy was consistent with a liberal approach. Postoperative complications in a fluid liberal setting are common.

This study justified the need for interventions to seek to minimize complications in our institution. Our observations provide sufficient data to design this interventional studies to decrease the incidence of complications in our institution.

Table 1: Summary of major and minor complication rates in patients undergoing colorectal and non-colorectal major abdominal procedures

	All operations	Colorectal	Non-colorectal	*p*
**Patients with at least one complication**	57 (57%)	28 (43%)	29 (82%)	0.003
**Patients with major complications**	32 (32%)	14 (21%)	18 (51.4%)	0.004
**Patients with minor complications**	52 (52%)	27 (41.5%)	25 (71.4%)	0.009
**Number of overall minor complications**	104	46	58	0.003
**Number of overall major complications**	63	23	40	0.012
**Major cardiac complications***	10	4	6	0.78
**Respiratory complications §**	30	11	19	0.001
**Tissue healing complications ¶**	20	8	12	0.012
**Total hospital days - mean (SD)**	18 (30)	12	30	0.004

* pulmonary oedema causing sudden death or CT/scintigraphic changes, myocardial infarction (ECG changes and myocardial enzyme elevation: cTropI>.06), ventricular arrhythmia (ECG changes/medical treatment or cardioversion), heart rate <50b/min requiring medical treatment/pacing)§ pulmonary embolism (causing sudden death or CT/scintigraphic changes), pneumonia (elevated temperature with radiographic changes), pulmonary congestion (shortness of breath, crepitations & medical treatment), pneumothorax (requiring drainage)

¶ anastomotic leakage, necrosis of stoma, superficial wound infection (surgical evacuation of pus or haematoma/secondary suture)

The authors then evaluated whether an Enhanced Recovery After Surgery program (ERAS) reduced length of hospital stay following uncomplicated pancreaticoduodenectomy[18]. We hypothesized that patients undergoing uncomplicated pancreaticoduodenectomy treated by an Enhanced Recovery After Surgery (ERAS) program would have a shorter length of hospital stay compared to those managed by standard care. Patients without surgical or medical complications following pancreaticoduodenectomy managed by ERAS or standard

programs between 2005 and 2011 were identified and prognostic predictors for length of hospital stay determined. Forty-one patients treated by pancreaticoduodenectomy had no medical or surgical complications during this period. Of these patients, 20 underwent ERAS program compared to 21 who underwent standard care. Median postoperative length of stay was shorter in the ERAS group (8 days, range: 7-16 days) *versus* 14 days, range: 8-29 days; P<0.001). There were three readmissions in the ERAS program related to abdominal pain and none in the standard group. The overall length of stay, accounting for readmissions, still remained significantly shorter in the fast track recovery program group (median 9 days, range: 7-17 days *versus* median14 days, range: 8-29 days; P<0.001). Patients in the standard group were more likely to have a feeding jejunostomy tube (P<0.001), pylorus preserving procedure (P=0.001) and a nasogastric tube in place longer than 24 hours postoperatively (P<0.001). There were no significant differences in discharge destination between groups. On multivariate analysis, the only factor independently associated with postoperative discharge by day 8 was ERAS program (OR: 37.1, 95% CI: 4.08-338;P<0.001). We concluded that ERAS program achieved significantly shorter length of stay following uncomplicated pancreaticoduodenectomy.

Table 2. Austin Hospital Data: Factors associated with discharge following pancreaticoduodenctomy by post-operative day 8

	8 Days or less LOS (n=14)	More than 8 days LOS (n=27)	Univariate		Multivariate	
			Odds ratio (Confidence interval)	Difference (p value)	Odds ratio (Confidence interval)	Difference (p value)
**Demographics**						
Male gender	9(64%)	16(59%)	1.2(0.3-4.7)	0.754		
BMI ≥ 30	1(7%)	5(5%)	1.4(0.8-24.2)	0.823		
Age ≥ 70	5(36%)	6(22%)	1.9(0.5-8.1)	0.355		
Pre-operative diabetes	6(43%)	4(15%)	4.3(1.0-19.3)	0.064	3.1(0.5-21.7)	0.251
ASA II	3(21%)	11(41%)	0.4(0.1-1.8)	0.305		
Biliary stent	6(43%)	4(15%)	4.3(1.0-19.3)	0.064	1.9(0.3-12.8)	0.528
Bilirubin ≥ 60 μmol/l)	6(43%)	6(22%)	2.6(0.7-10.6)	0.168		
Albumin < 30 g/l)	2(14%)	5(19%)	0.4(0.1-4.4)	0.733		
Epidural anesthesia	11(79%)	16(59%)	2.5(0.6-11.1)	0.216		
**Pathology**						
Malignancy	12(86%)	22(82%)	1.4(0.2-8.1)	0.733		
Positive resection margin	4(29%)	5(19%)	1.8(0.4-8.0)	0.461		
**Operative details**						
Time ≥ 8 hours	2(14%)	5(19%)	0.4(0.1-4.4)	0.733		
Blood loss ≥ 600ml	6(43%)	6(22%)	2.6(0.7-10.6)	0.168		
Blood transfusion intraoperative	1(7%)	6(22%)	0.3(0-2.5)	0.389		
Classic Whipple procedure	13(92%)	19(70%)	5.4(0.6-49.2)	0.131		
Feeding jejunostomy tube	0(0%)	14(45%)	1.6(1.2-2.1)#	0.017*	0.0(N/A)	0.99
**Post-operative details**	2(14%)	6(22%)	0.6(0.1-3.4)	0.543		
**Fast-track recovery**	**13(93%)**	**7(26%)**	**37(4.1-338.1)**	**<0.001***	**37(4.1-338.1)**	**0.001**
Blood transfusion post-operative						
Negative fluid balance day 1	1(7%)	2(7%)	1.0(0.1-11.6)	0.975		
Negative fluid balance day 2	8(57%)	7(26%)	3.8(1.0-14.9.)	0.049*	0.5(0.1-3.7)	0.510
Negative fluid balance day 3	9(64%)	11(41%)	2.6(0.7-10.0)	0.153		

LOS – Post-operative length of stay; ASA – American society of anesthesiologists; DGE – delayed gastric emptying; BMI – body mass index; *p≤0.05; # Odds ratio for absence of feeding jejunostomy, Chi-Square/Fisher's exact test

Table 3: Austin data. Post-operative and outcome details of patients undergoing uncomplicated pancreaticoduodenal resection managed by fast-track and standard protocols

	Overall (n=41)	Fast-track group (n=20)	Standard group (n=21)	Difference (p value)
Days in SICU	1(1-13)	1(1-3)	1(1-13)	0.633
Nasogastric removal day 1 post surgery	19(48%)	19(95%)	0(0%)	<0.001*
Discharged home	36(88%)	18(90%)	18(86%)	0.675
Blood transfusion	8(20%)	2(10%)	6(28%)	0.238
Negative fluid balance day 1 post surgery	3(7%)	2(10%)	1(5%)	0.481
Negative fluid balance day 2 post surgery	15(37%)	13(65%)	2(10%)	<0.001*
Negative fluid balance day 3 post surgery	20(50%)	11(55%)	9(42%)	0.437
Readmissions	3(7%)	3(15%)	0(0%)	0.107
Initial post-operative length of stay (days)	10(7-29)	8(7-16)	14(8-29)	<0.001*
Overall length of stay including readmissions (days)	10(7-29)	9(7-16)	14(8-29)	<0.001*

SICU – Surgical intensive care unit; Missing values – nasogastric tube removal day 1 post surgery =1;
*p≤0.05 Chi-Square/Fisher's exact test

Finally we evaluated whether liberal fluid intervention and positive fluid balance affect length of hospital stay following pancreaticoduodenectomy (Whipple's Procedure)[19]. The rationale for this study is that the effects on detailed fluid intervention on perioperative complications and length of hospital stay (LOS) after pancreaticoduodenectomy have not been reported. We hypothesized that patients undergoing pancreaticoduodenectomy treated with a liberal fluid regime would have a longer LOS compared to those managed with a restrictive regime. We conducted a retrospective analysis of patients undergoing pancreaticoduodenectomy at Austin Hospital. Demographic data, operative details, detailed fluid prescription, complications and outcomes were retrieved from medical records. Prognostic predictors for LOS were determined. Data for 150 consecutive patients undergoing pancreaticoduodenectomy between 2004 - 2012 was collected. Mean age: 66 years; Male gender: 59%, Mean weight: 74kg, ASA Class 3: 75%. Average volume of intravenous fluid administered on postoperative days (POD) 1 to 3 was 3.0L (range: 0.9-14.1), 2.1L (range: 0.3-6.1), and 1.7L (range: 0-6.0) respectively. 50 patients (33%) experienced at least one major complication; 66 patients (44%) at least one minor complication. Common complications: sepsis (22%), anastomotic leak (20%), pneumonia (17%), delayed gastric emptying (17%), myocardial infarction (8%), pulmonary oedema (6%). 30-day mortality: 2%. Median LOS: 17 days (range: 7-140 days).

We concluded that patients experiencing complications received more fluid intraoperatively, and on POD 1-3 (P=0.02). Positive fluid balance was a strong independent predictor of hospital morbidity. On multivariate analysis, factors independently associated with a LOS of <14 days were complications (OR 0.1, 95% CI: 0.0-0.3;P<0.001) and >3L fluid on POD 1 (OR 3.0, 95% CI: 1.1-8.3;P<0.003).

This study demonstrated that liberal perioperative fluid intervention and a positive fluid balance adversely affect LOS stay following pancreaticoduodenectomy.

These studies have provided important pilot data to plan and power the current proposed study.

3. Study Objectives

3.1 Hypothesis

Patients undergoing pancreaticoduodenectomy (Whipple's procedure) managed by intraoperative goal directed therapy with the Flotrac/Vigileo device^{TM} and will have a shorter length of hospital stay with fewer post operative complications compared to patients managed by standard care.

3.2 Study Aims

The study aims are to evaluate if undergoing pancreaticoduodenectomy (Whipple's procedure) managed by intraoperative goal directed therapy with the Flotrac/Vigileo device^{TM} will have a shorter length of hospital stay with fewer post operative complications compared to patients managed by standard care. The study will also evaluate perioperative fluid interventions, haemodynamics, use of inotropes, and duration of stay in the intensive care unit.

3.3 Outcome Measures

Primary outcome:

- Length of hospital stay

Secondary outcomes

- Intraoperative haemodynamic data including cardiac output, cardiac index, mean arterial pressure, stroke volume variation, systemic vascular resistance and heart rate: all downloaded directly from the Flotrac/Vigileo device^{TM})
- Major morbidity (pulmonary congestion, pneumonia, heart failure, arrhythmias, acute kidney injury, intra-abdominal collections, haemorrhage, pancreatic anastomotic leakage, and delayed gastric emptying)
- Blood transfusion requirements
- Intraoperative fluid balances (type, amount)
- Postoperative fluid balances (type, amount)
- Use of inotropes (type, amount, duration)
- Duration of ICU/HDU stay

4. Study Design

4.1 Study Type & Design & Schedule

Design

A schematic overview of the study design is summarized in Figure 1.

This is a randomised multicenter controlled clinical trial. Patients will be randomized to standard current treatment of care, or standard current treatment of care with the addition of the Flotrac/Vigileo device™.

Standard of care for all patients

As per standard of care for all patients undergoing this procedure at our institution, prior to induction of anaesthesia, in the sitting position, 200-300ug of morphine will be deposited into the L3/4 intrathecal space using a strictly aseptic technique. The patient will then be placed supine on the operating table. Sequential compression stockings will be applied and meticulous attention to pressure care will taken using a gel mattress, gel pads for the arms and legs, and cottonwool padding under the hips, buttocks and sacral areas for additional pressure support.

Induction of anaesthesia will be achieved with a balanced technique of fentanyl 3 *u*g/kg IV, and propofol 3 mg/kg IV. After neuromuscular blockade with vecuronium 0.1 mg/kg IV, the trachea will be intubated. A 20G arterial line will be inserted into the radial artery and a triple lumen central venous catheter inserted into the right internal jugular vein using a ultrasound guided technique. A 16G nasogastric tube will be inserted. Dexamethasone phosphate 8 mg IV will be administered as part of routine antiemetic prophylaxis. Anaesthesia will be maintained with desflurane at inspired concentrations of 0.5-0.1 MAC, with a fractional inspired oxygen-air concentration of 0.5, and an infusion of remifentanil 0.1-0.3 *u*g/kg/min IV. Mechanical ventilation maintained end tidal pCO_2 between 35-40 mmHg.

Standard Monitoring

Standard monitoring for all patients undergoing pancreaticoduodenectomy included a 5-lead continuous electrocardiograph, pulse oximetry, capnography, urine output and core body temperature, invasive arterial monitoring and the insertion of a central venous catheter. Intra-operative normothermia will be maintained with warm fluids and a forced-air warming device will be used. A Flotrac device FloTrac/Vigileo™ (Edwards Lifesciences, Irvine, CA) will be connected to the radial line for advanced haemodynamic monitoring. Systolic blood pressure will be maintained within 20% of the pre-operative value. In keeping with our institutions antibiotic prophylaxis protocol, at induction of anesthesia all patients received Ampicillin 1 g IV, Ceftriaxone 1 g IV and Metronidazole 500 mg IV. Evidence of adequate end-organ perfusion was guided by serum lactate, base deficit, ScVO2 >70, Bispectral index >40, and normal ST-segments.

Fluid intervention, and use of inotropes and vasoconstrictors will guided by an advanced hemodynamic algorithm described in Figure 1. An actrapid insulin infusion at 4-8 units/hr was used to maintain blood glucose levels between 8-10 mmol/L. One hour prior to completion of surgery, tramadol 3 mg/kg IV and paracetamol 1 g IV was administered, and a continuous ketamine infusion started at 2 mg/hr IV. Once the surgery was complete, and the abdomen being closed, fentanyl 150 ug IVI was administered, and a continuous fentanyl infusion was commenced at 10 ug/hr IVI. The remifentanil infusion and desflurane anestheisa were discontinued and after the abdomen was closed, and the patient was extubated within 6 minutes.

All patients will be ventilated at 8 mL/kg of ideal body weight and their respiratory rate will be adjusted such that they have minute ventilation of approximately 7–8 L/min with an I:E ratio of 1:2.

Figure 1: Schematic overview of study design

Participant Consent in pre-admission anaesthesia clinic
N = 52

Inclusion criteria

- ✓ Adult patients
- ✓ Elective open pancreaticoduodenectomy

Exclusion criteria

- Age less <18 years
- Abnormal pre-operative coagulopathy: INR > 1.4, platelet count < 75 x 10^9/l
- Severe hepatic insufficiency (bilirubin > 30umol/L, ALP > 300iu/L, ALT > 50iu/L, albumin < 25g/dL)
- Severe renal impairment: serum creatinine > 250*u*mmol/l
- ASA status > physical IV or V

RANDOMISATION

Control Group
N=26

To receive the standard surgical and anaesthesia care with an enhanced Enhanced Recovery After Surgery Program (ERAS)
+
Edward Lifesciences Flotrac™ monitoring connected on insertion of arterial line but screen BLINDED to anaesthetist
FloTrac stppped immediately after surgery complete

Goal Directed Therapy Group (GDT)
N=26

To receive the standard surgical and anaesthesia care with an enhanced Enhanced Recovery After Surgery Program (ERAS)
+
GDT with Edward Lifesciences Flotrac™ monitoring to commence on insertion of arterial line and stppped immediately after surgery complete

Intraoperatively: all patients to receive standard surgical and anaesthesia care

- ✓ ECG
- ✓ Arterial line
- ✓ Central venous catheter
- ✓ Urinary catheter
- ✓ BIS monitor i.e. depth of anaesthesia monitoring
- ✓ Fluid warmer and bear hugger
- ✓ Fluid administration and delivery of anaesthesia as per routine anaesthesia care

Postoperative Care

- As per standard surgical protocol
- All patients to receive ERAS program
- The anaesthetist will have no influence over any aspect of postoperative care

Primary endpoint:

- ✓ Length of hospital stay

Secondary endpoints:

- ✓ Major morbidity e.g. postoperative medical and surgical complications e.g. pulmonary congestion, pneumonia, heart failure, arrhythmias, acute kidney injury, intra-abdominal collections, haemorrhage, pancreatic anastomotic leakage, and delayed gastric emptying
- ✓ Intraoperative haemodynamic data e.g. cardiac output, cardiac index, stroke volume variation, MAP, SVR & HR
- ✓ Blood transfusion requirements
- ✓ Intraoperative fluid balances (type, amount)
- ✓ Postoperative fluid balances (type, amount)
- ✓ Use of inotropes (type, amount, duration)
- ✓ Duration of ICU/HDU stay

Other data collected: Daily body weight; Pain Scores; Routine biochemistry and haematology; Drain output; Hospital discharge; Readmission with 30 days; Intraoperative and 30-day mortality

Figure 2: The fluid management protocol used for the GDT group

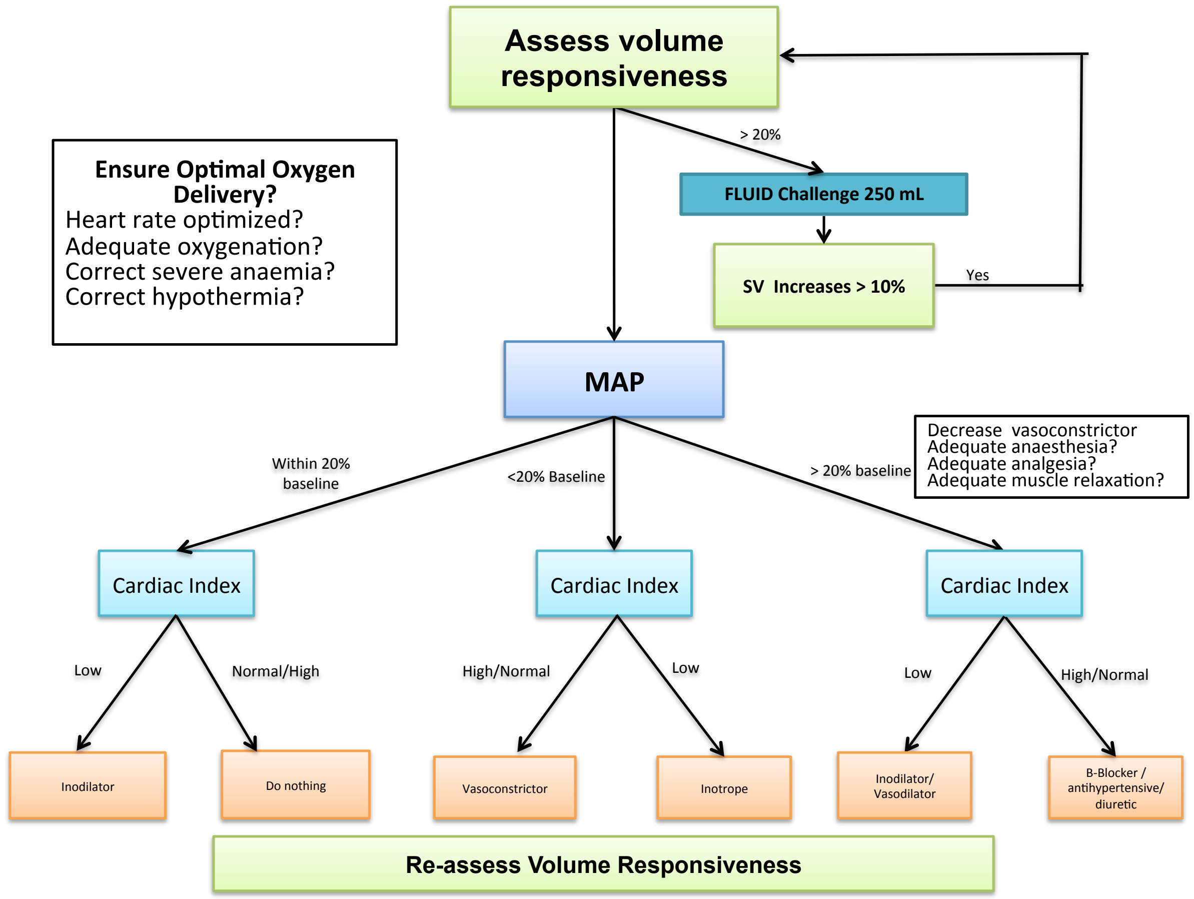

Table 4: Summary of the enhanced recovery pathway protocol for all patients undergoing major pancreatic surgery in our institution.

**Before surgery**	- Preoperative multidisciplinary evaluation<br>- Optimization of preoperative comorbidities<br>- Education of patients and families and informed consent<br>- Routine preoperative biochemistry, haematology, coagulation tests, CXR, ECG<br>- Daily weight
**Anaesthesia**	- Single shot intrathecal morphine for postoperative analgesia<br>- Insertion of Flotrac/Vigileo™ haemodynamic monitoring device<br>- Antibiotics: Ampillicin 1 g, Ceftriaxone 1 g, Flagyl 500 mg<br>If penicillin allergy: Vancomycin 1g.<br>- Intraoperative supplementation of magnesium and potassium<br>- Intraoperative prophylactic anticoagulation 2 hours after insertion of intrathecal morphine<br>- Insertion nasogastric tube<br>- Maintenance of anaesthesia and analgesia: Remifentanil and Desflurane<br>**One hour prior to completion of surgery:**<br>- Commencement of intravenous fentanyl infusion at 10ug/hr<br>- Commencement of intravenous Ketamine infusion at 2mg/hr

	- Paracetamol 1g, Tramadol 3 mg/kg<br>- Consider non steroidal anti-inflammatory
**Recovery and Day of Surgery**	**Analgesia**<br>- PCA fentanyl (20ug/bolus,5 min lockout)<br>- Continue fentanyl infusion (10ug/hr)<br>- Continue ketamine infusion (2mg/hr)<br>- Strict QID paracetamol<br>- prn tramadol, low dose ketamine infusion (2mg/hr)<br>**Fluid intervention**<br>- 12 hourly balanced crystalloid maintenance fluid therapy<br>- 4% albumex 250 mls prn for filling<br>**Other**<br>- Postoperative haemodynamic optimization (Figure 1)<br>- Strict TDS metoclopramide 15 mg IVI<br>- Potassium and magnesium supplementation<br>- Continue triple antibiotics for 24 hours<br>- Pantroprazole 40 mg daily<br>- Antithrombotic prophylaxis (clexane or heparin)
**Day 1**	**Analgesia**<br>- Stop Fentanyl background infusion<br>- Continue PCA fentanyl (20ug/bolus,5 min lockout)<br>- Continue ketamine infusion (2mg/hr) if no side effects<br>- Strict QID paracetamol<br>- prn tramadol<br>**Fluid intervention**<br>- Reduce maintenance fluid therapy to 83mls/hr if on ward<br>- 4% albumex 250 mls prn for filling<br>**Other**<br>- Daily weight<br>- Strict TDS metoclopramide 15 mg IVI<br>- Potassium and magnesium supplementation<br>- Continue triple antibiotics for 24 hours<br>- Pantroprazole 40 mg daily<br>- Remove NGT unless output > 300mls/day<br>- Physiotherapy: early mobilization TDS<br>- Antithrombotic prophylaxis
**Day 2**	**Analgesia**<br>- Continue PCA fentanyl (20ug/bolus,5 min lockout)<br>- Stop ketamine infusion<br>- Strict QID paracetamol<br>- prn tramadol<br>**Fluid intervention**<br>- Aim for neutral fluid balance<br>- Reduce maintenance fluid therapy to 42 mls/hr<br>- Commence oral sips<br>- 4% albumex 250 mls prn for filling<br>**Other**<br>- Daily weight<br>- Strict TDS metoclopramide 15 mg IVI<br>- Potassium and magnesium supplementation

	- Pantroprazole 40 mg daily<br>- Use of diuretic if positive fluid balance (furosemide 10-20mg)<br>- Strict Metoclopramide 15mg TDS<br>- Pantroprazole 40 mg daily<br>- Continue antithrombotic prophylaxis<br>- Removal of central venous catheter<br>- Removal of urinary catheter<br>- Physiotherapy: early mobilization TDS
**Day 3**	**Analgesia**<br>- Stop fentanyl PCA<br>- Start oxycodone (IR) 10 mg 4 hourly<br>- Strict QID paracetamol<br>- prn tramadol<br>**Fluid intervention**<br>- Aim for neutral fluid balance<br>- Use of diuretic if positive fluid balance (frusemide 10-20mg)<br>- Stop intravenous maintenance fluids<br>- Commence oral sips<br>- 4% albumex 250 mls prn for filling<br>**Other**<br>- Daily weight<br>- Strict TDS metoclopramide 15 mg IVI<br>- Potassium and magnesium supplementation<br>- Pantroprazole 40 mg daily<br>- Continue antithrombotic prophylaxis<br>- Removal of central venous catheter<br>- Removal of urinary catheter<br>- Physiotherapy: early mobilization TDS<br>- Coloxyl 100mg BD
**Day 4**	**Fluid intervention**<br>- Advancing from soft diet to small meal<br>- Daily weight<br>- Use of diuretic if positive fluid balance (furosemide 10-20mg)<br>**Analgesia**<br>- Continue oral oxycodone IR<br>- prn paracetamol and tramadol<br>**Other**<br>- Stop metoclopramide<br>- Continue antithrombotic prophylaxis<br>- Physiotherapy therapy<br>- Coloxyl 100mg BD<br>- Start Creon 25,000 units x1 with meals (2 tablets if total pancreatectomy with main meals) and 1 with snacks
**Day 5**	**Fluid intervention**<br>- Advance diet<br>- Use of diuretic if positive fluid balance (frusemide 10-20mg)<br>**Analgesia**<br>- Continue oral oxycodone IR<br>- prn paracetamol and tramadol<br>- Continue antithrombotic prophylaxis<br>- Consider removing wound drains

**Day 6**	- Full ward diet<br>- Daily weight<br>- Continue oral oxycodone IR prn or tramadol<br>- prn paracetamol<br>- Consider hospital discharge
**Day 7**	- Discharge

4.2 STANDARD CARE AND ADDITIONAL TO STANDARD CARE PROCEDURES

**Standard Care Procedures**		
**Procedure**	**Time/Visit**	**Dosage**
Arterial Line insertion	Intra-operatively	N/A
Central Venous Line Insertion	Intra-operatively	N/A
Admission to High Dependency Unit	Post-operatively	

**Additional To Standard Care**		
**Procedure**	**Time/Visit**	**Dosage/Volume**
Use of Flotrac device	Intra-operatively	N/A

4.3 RANDOMISATION

Participants will be randomly assigned to one of two groups using a computer generated random number allocation system with permuted blocks.

Patients will be randomised to:

1. **Control Group:** to receive the standard perioperative ERAS care

2. **Experimental Group:** to receive standard perioperative ERAS care and additional haemodynamic monitoring using the FloTrac/Vigileo™ (Edwards Lifesciences, Irvine, CA).

Both groups of patients will undergo an ERAS program, which is standard of care in our institution. In keeping with standard practices at all the recruiting centres, both groups of patients will undergo preoperative multidisciplinary team assessment, where surgeon, oncologist, radiation oncologist, radiologist, anaesthetist, nutritionist, and others, will ensure that patients are optimized for their surgery.

Blinding

Surgical teams, intraoperative and postoperative nursing staff and patients will be blinded to group assignment, but the anaesthetist will be aware of group designation. Both groups of patients will have the Flotrac/Vigileo device™ connected after insertion of the arterial line. The arterial catheters will be connected to the cardiac output device transducer system and haemodynamic data from the Flotrac device will be collected throughout surgery in all patients.

All transducers will be zeroed to atmospheric pressure at the level of the right atrium. Control patients will have fluid management and inotropic use guided by the routine cardiovascular monitoring in place i.e. arterial line, and central venous catheter, which will be at the discretion of the anaesthetist, who will be blinded to Flotrac data. The Control group anaesthetist will be allowed to have the Flotrac haemodynamic data unblinded if needed for clinical decision making but patients will be removed from analysis if this occurs.

In contrast, GDT patients were managed by an SVV guided protocol to maintain SVV < 20%. The fluid management protocol used for the GDT group is shown above in Figure 2. This percentage of SVV is frequently used in our institution as a target for patients undergoing this procedure. For the GDT group, if SVV remains above 20% for at least 2 min, then a 250 mL bolus of balanced crystalloid (Hartmanns solution or Plasmalyte) or colloid fluid (Albumen) will be given. The SVV will be assessed every 20 seconds via the FloTrac/Vigileo proprietary algorithm. Similar to other GDT studies, the preferred colloid will be albumin secondary to the known effects of improved intravascular repletion and intravascular half life and the theoretical benefit of prolonged stroke volume optimization. In both groups, the administration of blood products will be at the discretion of the anaesthetist, as clinically indicated. There will be no standard protocol for fluid maintenance infusion for either group.

The anaesthetist will have no influence over any aspect of postoperative care management. All clinicians and nursing staff in charge of postoperative care will be blinded to the allocation of patients.

The Flotrac system will be connected to the patient prior to surgical incision and discontinued as soon as the surgery is complete.

4.4 STUDY METHODOLOGY

Haemodynamic data FloTrac/Vigileo device will be downloaded directly from the device via a inbuilt USB port 3-hours postoperatively. This data will include:

- Cardiac output
- Cardiac index
- Stroke volume variation
- Mean arterial pressure
- Heart rate
- Systemic vascular resistance

In addition the following data will be collected:

- Demographic characteristics
- Duration of surgery
- Intraoperative blood transfusion requirements
- Intraoperative fluid balances (type, amount)

- Postoperative fluid balances (type, amount)
- Use of inotropes (type, amount, duration)
- Duration of ICU/HDU stay
- Complications
- Daily body weight
- Pain Scores
- Routine biochemistry and haematology
- Drain output
- Hospital discharge
- Readmission with 30 days
- Intraoperative and 30-day mortality

5. Study Population

5.1 Recruitment Procedure

All patients undergoing pancreaticoduodenectomy will be evaluated preoperatively at the anaesthesia pre-admissions clinic at least 1-2 weeks prior to surgery. Patients will be identified for study entry by the investigators or an anaesthetist or research co-ordinator acting on behalf of the principle investigators by surveillance of patients in the pre-admissions clinic. Patients will be identified from their preoperative medical records and surgical notes.

Inform consent will then be obtained.

5.2 Inclusion Criteria

✓ All patients > 18 years undergoing pancreaticoduodenectomy

5.3 Exclusion Criteria

✓ Age less than 18 years
✓ Abnormal pre-operative coagulopathy: INR > 1.5, platelet count < 75 x 10^9/l
✓ Severe hepatic insufficiency (bilirubin > 30umol/L, ALP > 300iu/L, ALT > 50iu/L, albumin < 25g/dL, INR > 1.5)
✓ Severe renal impairment: serum creatinine > 250*u*mmol/l
✓ American Society of Anesthesiologists (ASA) physical status IV or V

5.4 Consent

A thorough assessment of the participant's competence and capacity to make a valid informed decision will be made by one of the study investigators prior to the patient being recruited. All patients will be deemed competent if they:

1. are able to comprehend and retain information relevant to making the decision
2. understand the information and implications of the decision
3. are able to weigh the information in the balance and arrive at a decision

6. PARTICIPANT SAFETY AND WITHDRAWAL

6.1 RISK MANAGEMENT AND SAFETY

Recruitment for this study will in no way compromise standard of care for any patient undergoing this procedure. Both groups of patients will undergo an ERAS program, which is standard of care in our institutions for all patients undergoing pancreaticoduodenectomy.

Control patients will have fluid management and inotropic use guided by the routine cardiovascular monitoring in place i.e. arterial line, and central venous catheter, which will be at the discretion of the anaesthetist, who will be blinded to Flotrac data. To ensure the most stringent safety of the study, the anaesthetist will be allowed to have the Flotrac haemodynamic data unblinded if needed for clinical decision-making but patients will be removed from analysis if this occurs.

6.2 HANDLING OF WITHDRAWALS

Participants may withdraw from the study at ant point for the following reasons: participant has chosen to withdraw from the study, protocol violation, or if the control group anaesthetist is unblinded to the Flotrac haemodynamic data. In these circumstances, the participants will be removed from analysis.

6.3 REPLACEMENTS

From our experiences with the 3 pilot studies outlined above, withdrawn participants will be not replaced in the study. This will not have any statistical significance of the sample size for the study.

7. STATISTICAL METHODS

7.1 SAMPLE SIZE ESTIMATION & JUSTIFICATION

We will recruit 52 patients in total, 26 patients in the GDT group and 26 patients in the standard group.

This is in keeping with realistic power calculations used in other studies evaluating the use of GDT in patients undergoing major abdominal surgery[4,6,20]. Canesson's group in the US detect a 3-day mean length of stay difference between the two groups, with a standard deviation of 3 days in each group, a two tailed alpha of 0.05 and power of 0.80, and calculated that a minimum of 17 subjects were required in each group[20]. The assumed that a similar sample size would be needed to detect a similar difference in time to return of GI function. Also, since they expected similar results between arterial pressure waveform analysis technology used for their study and the oesophageal Doppler technology used for other referenced studies, it was felt that this estimation was appropriate.

7.2 POWER CALCULATIONS

Sample size for the study was calculated based on our pilot data evaluating patients undergoing pancreaticoduodenectomy with an ERAS program at Austin hospital. With an median length of hospital stay of 16 days, and a SD of 4 days, if we were to demonstrate a mean difference between the control group and the GDT group of 2 days, with a power value

of 80%, we require a minimum of 21 patients to be recruited into each group. We will recruit 5 additional patients in each group, given the large SD.

Summary of power calculations for this study

Confidence Interval (2-sided)		95%		
Power		80%		
Ratio of sample size (Group 2/Group 1)		1		
	**Group 1**		**Group 2**	**Mean difference[1]**
Mean	16			2.5
Standard deviation	4		0.0	
Variance	0.0		0.0	

Sample size of Group 1	21	
Sample size of Group 2	21	
Total sample size	42	

[1] Mean difference= (Group 1 mean) - (Group 2 mean)

RESULTS FROM OPENEPI, VERSION 2, OPEN SOURCE CALCULATOR--SSMEAN
http://www.openepi.com/OE2.3/SampleSize/SSMean.htm
Source file last modified on 09/21/2010 13:10:35

7.3 STATISTICAL METHODS TO BE UNDERTAKEN

Statistical analysis will be performed using computerized software (SPSS for Windows version 12.0). For data that was non-normally distributed a Mann–Whitney test was used and normally distributed data were compared using the Student T test.Ordinal and nominal data were compared using Chi square analysis. A p-value <0.05 will be considered significant. Standard statistical tests will be used, and an independent statistician blinded to the randomisation will undertake all statistical analyses.

8. DATA SECURITY & HANDLING

8.1 DETAILS OF WHERE RECORDS WILL BE KEPT & HOW LONG WILL THEY BE STORED

The information to be stored during and after this RCT will be in the form of password protected electronic computer files on a dedicated hard drive in the department of

anaesthesia. Data will be password protected on a dedicated storage file set up by the Information Technology department at Austin Hospital. Only the principle investigators will have access to this electronic database. Any paper data will be locked in dedicated research storage drawers in the department of anaesthesia.

All data records both electronic and paper will be retained for 15 years prior to destruction/shredding in accordance with Good Clinical Practice for clinical trials. Information will be disposed of securely by shredding of paper documents and permanent erasure of electronic data. Any back up in the form of CD disc/DVD's will be destroyed.

8.2 CONFIDENTIALITY AND SECURITY

Data will be password protected on a dedicated storage file set up by the Information Technology department at Austin Hospital. Only the principle investigators will have access to this electronic database. Any paper data will be locked in dedicated research storage drawers in the department of anaesthesia.

Study organization

This study will be managed and coordinated by a team of clinician researchers with experience in the conduct of perioperative clinical trials. The Department of Anaesthesia Research Group will centrally manage the project.

Timelines and milestones

Date	Anticipated Project Milestone
April 2013	Ethics application and subsequent regulatory requirements
June 2013	Commence recruitment
May 2015	Database lock, data analysis and initial results
Dec 2015	Publication

<u>Detailed Budget</u>

Item	Year 1
Cost of FloTrac Devices @$150each<br>X 52devices	7770

Maintenance

Item	Year 1
Computer consumables (printing, paper, ink)	500
Professional statistical consultation	2,000

Other Items

Item	Year 1
Fee to Human Research Ethics Committees: Investigator initiated	

Total (AUD)	$10,270

9. Appendix

List of Attachments included:

Document Name	Version Number	Date (e.g., 18 January 2012)
Edwards Lifesciences EV 1000 Clinical Platform	1	21 Jan 2013
Edwards Lifesciences 3rd generation SOFTWARE	1	21 Jan 2013
Edwards Lifesciences Flotrac Algorithm	1	21 Jan 2013

10. References

1. NHS Technology Adoption Centre- Intraoperative Fluid Management Technologies Adoption Pack. http://www.ntac.nhs.uk/NewsAndEvents/Intraoperative_Fluid_Management_Adoption_Pack_Published.aspx (Accessed Dec 2012)

2. Kumar A, Anel R, Bunnell E, Habet K, Zanotti S, Marshall S, Neumann A, Ali A, Cheang M, Kavinsky C, Parrillo JE. Pulmonary artery occlusion pressure and central venous pressure fail to predict ventricular filling volume, cardiac performance, or the response to volume infusion in normal subjects. Crit Care Med. 2004 Mar;32(3):691-9.

3. Michard F, Teboul JL. Predicting fluid responsiveness in ICU patients: a critical analysis of the evidence. Chest 2002 Jun;121(6):2000-8.

4. Osman D, Ridel C, Ray P, Monnet X, Anguel N, Richard C, Teboul JL.
Cardiac filling pressures are not appropriate to predict hemodynamic response to volume challenge. Crit Care Med. 2007 Jan;35(1):64-8.

5. Marik PE, Monnet X, Teboul JL. Hemodynamic parameters to guide fluid therapy.
Ann Intensive Care. 2011 Mar 21;1(1):1.

6. Abbas SM, Hill AG. Systematic review of the literature for the use of oesophageal Doppler monitor for fluid replacement in major abdominal surgery. Anaesthesia. 2008;63(1):44–51.

7. Hamilton MA, Cecconi M, Rhodes A. A systematic review and meta-analysis on the use of preemptive hemodynamic intervention to improve postoperative outcomes in moderate and high-risk surgical patients. Anesth Analg. 2011 Jun;112(6):1392-402.

8. Giglio M, Dalfino L, Puntillo F, Rubino G, Marucci M, Brienza N.
Haemodynamic goal-directed therapy in cardiac and vascular surgery. A systematic review and meta-analysis. Interact Cardiovasc Thorac Surg. 2012 Nov;15(5):878-87.

9. Giglio MT, Marucci M, Testini M, Brienza N. Goal-directed haemodynamic therapy and gastrointestinal complications in major surgery: a meta-analysis of randomized controlled trials. Br J Anaesth. 2009 Nov;103(5):637-46.

10. Dalfino L, Giglio MT, Puntillo F, Marucci M, Brienza N. Haemodynamic goal-directed therapy and postoperative infections: earlier is better. A systematic review and meta-analysis. Crit Care. 2011 Jun 24;15(3):R154. doi: 10.1186/cc10284. Review.

11. Brienza N, Giglio MT, Marucci M, Fiore T.Does perioperative hemodynamic optimization protect renal function in surgical patients? A meta-analytic study. Crit Care Med. 2009 Jun;37(6):2079-90. doi: 10.1097/CCM.0b013e3181a00a43. Review.

12. Phan TD, Ismail H, Heriot AG, Ho KM. Improving perioperative outcomes: fluid optimization with the esophageal Doppler monitor, a metaanalysis and review.
J Am Coll Surg. 2008 Dec;207(6):935-41.

13. Gan TJ, Soppitt A, Maroof M, el-Moalem H, Robertson KM, Moretti E, Dwane P, Glass PS. Goal-directed intraoperative fluid administration reduces length of hospital stay after major surgery. Anesthesiology. 2002;97(4):820–6.

14. Noblett SE, Snowden CP, Shenton BK, Horgan AF. Randomized clinical trial assessing the effect of Doppler-optimized fluid management on outcome after elective colorectal resection. Br J Surg. 2006;93(9):1069–76.

15. Rhodes A, Cecconi M, Hamilton M, Poloniecki J, Woods J, Boyd O, Bennett D, Grounds RM. Goal-directed therapy in high-risk surgical patients: a 15-year follow-up study.
Intensive Care Med. 2010 Aug;36(8):1327-32. doi: 10.1007/s00134-010-1869-6. Epub 2010 Apr 8.

16. Lees N, Hamilton M, Rhodes A. Goal-directed therapy in high risk surgical patients.
Crit Care. 2009;13(5):231. doi: 10.1186/cc8039. Epub 2009 Oct 26. Review.

17. Warrillow SJ, Weinberg L, Parker F, Calzavacca P, Licari E, Aly A, Bagshaw S, Christophi C, Bellomo R. Perioperative fluid prescription, complications and outcomes in major elective open gastrointestinal surgery. Anaesth Intensive Care. 2010 Mar;38(2):259-65.

18. Nikfarjam M, Weinberg L, Low N, Fink MA, Muralidharan V, Houli N, Starkey G, Jones R, Christophi C. A fast track recovery program significantly reduces hospital length of stay following uncomplicated pancreaticoduodenectomy. JOP. 2013 Jan 10;14(1):63-70. doi: 10.6092/1590-8577/1223.

19. Weinberg L, Christophi C, McNicol L, Nikfarjam M. Liberal fluid intervention and positive fluid balance affect length of hospital stay following pancreaticoduodenectomy (Whipple's Procedure). Manuscript in progress. Abstract Submitted to Australian & New Zealand College of Anaesthetist Annual Scientific Meeting, 2013

20. Ramsingh DS, Sanghvi C, Gamboa J, Cannesson M, Applegate RL 2nd.J Clin Monit Comput. 2012 Dec 22. [Epub ahead of print] Outcome impact of goal directed fluid therapy during high risk abdominal surgery in low to moderate risk patients: a randomized controlled trial. J Clin Monit Comput. 2012 Dec 22. [Epub ahead of print]
